# The low-temperature germinating spores of the thermophilic *Desulfofundulus* contribute to an extremely high sulfate reduction in burning coal seams

**DOI:** 10.3389/fmicb.2023.1204102

**Published:** 2023-09-15

**Authors:** Olga V. Karnachuk, Igor I. Rusanov, Inna A. Panova, Vitaly V. Kadnikov, Marat R. Avakyan, Olga P. Ikkert, Anastasia P. Lukina, Alexey V. Beletsky, Andrey V. Mardanov, Yuri V. Knyazev, Mikhail N. Volochaev, Nikolai V. Pimenov, Nikolai V. Ravin

**Affiliations:** ^1^Laboratory of Biochemistry and Molecular Biology, Tomsk State University, Tomsk, Russia; ^2^Institute of Microbiology, Research Centre of Biotechnology of the Russian Academy of Sciences, Moscow, Russia; ^3^Institute of Bioengineering, Research Centre of Biotechnology of the Russian Academy of Sciences, Moscow, Russia; ^4^Kirensky Institute of Physics, Krasnoyarsk, Russia

**Keywords:** sulfate reduction, thermophiles, burning coal seams, *Desulfofundulus*, spores

## Abstract

Burning coal seams, characterized by massive carbon monoxide (CO) emissions, the presence of secondary sulfates, and high temperatures, represent suitable environments for thermophilic sulfate reduction. The diversity and activity of dissimilatory sulfate reducers in these environments remain unexplored. In this study, using metagenomic approaches, *in situ* activity measurements with a radioactive tracer, and cultivation we have shown that members of the genus *Desulfofundulus* are responsible for the extremely high sulfate reduction rate (SRR) in burning lignite seams in the Altai Mountains. The maximum SRR reached 564 ± 21.9 nmol S cm^−3^ day^−1^ at 60°C and was of the same order of magnitude for both thermophilic (60°C) and mesophilic (23°C) incubations. The *16S rRNA* profiles and the search for *dsr* gene sequences in the metagenome revealed members of the genus *Desulfofundulus* as the main sulfate reducers. The thermophilic *Desulfofundulus* sp. strain Al36 isolated in pure culture, did not grow at temperatures below 50°C, but produced spores that germinated into metabolically active cells at 20 and 15°C. Vegetative cells germinating from spores produced up to 0.738 ± 0.026 mM H_2_S at 20°C and up to 0.629 ± 0.007 mM H_2_S at 15°C when CO was used as the sole electron donor. The Al36 strain maintains significant production of H_2_S from sulfate over a wide temperature range from 15°C to 65°C, which is important in variable temperature biotopes such as lignite burning seams. Burning coal seams producing CO are ubiquitous throughout the world, and biogenic H_2_S may represent an overlooked significant flux to the atmosphere. The thermophilic spore outgrowth and their metabolic activity at temperatures below the growth minimum may be important for other spore-forming bacteria of environmental, industrial and clinical importance.

## 1. Introduction

Burning coal seams and waste piles are widespread throughout the world and are found in China, Russia, United States, South Africa, Australia, Germany, India, Indonesia and many other countries (Prakash and Gupta, 1998; Prakash et al., 1999; Stracher and Taylor, 2004; Kuenzer et al., 2007; Shan et al., 2019). Coal fires have been declared a global catastrophe, not only because of the huge loss of coal resources and land desertification, but also because of environmental problems such as the uncontrolled release of toxic fumes and greenhouse gases (Stracher, 2004). Gaseous compounds such as carbon-monoxide (CO), carbon-dioxide (CO_2_), methane (CH_4_), arsenic, fluorine and mercury have been discussed as the main problems associated with coal-burning fumes (Kuenzer et al., 2007; Pone et al., 2007; Song et al., 2020). So far, less attention has been paid to hydrogen sulfide emissions from burning coal seams. Garrison et al. (2017) reported a strong positive correlation between CO flux and H_2_S flux in a coal mine fire in Lotts Creek, Kentucky. Abnormally high concentrations of hydrogen sulfide have been reported in the Fukang mining area in western China (Xie et al., 2021). Coal contains sulfur in organic and inorganic forms (Calkins, 1994). Pyrite makes up the bulk of the inorganic sulfur in most coals. Sulfates, originated from the oxidation of S-rich coal-fire gases followed by subsequent evaporation, are formed in large quantities in burning coal piles (Pone et al., 2007; Kruszewski, 2013). The high amount of sulfate and the presence of potential electron donors such as CO and H_2_ in the burning seams make coal fire-associated heated rocks suitable biotopes for thermophilic sulfate reduction. However, the microbial sulfate reduction in burning coal seams or dumps remains unexplored.

We previously described a low-diversity microbial community dominated by *Firmicutes* (recently renamed *Bacillota*) associated with the burning lignite seams of the Taldy-Dyurgunskoye coalfield in the Altai Mountains (Kadnikov et al., 2018). Presumably, the thermophilic *Firmicutes* spores could have spread from their original thermal habitats by air and colonize burning coal seams rich in high-energy substrates such as H_2_ and CO. Subsequent regular site sampling revealed that H_2_S and CO were the main gases emitted from thermal biotopes, suggesting that microbial sulfate reduction may occur in the environment. This study reports extremely high rates of bacterial sulfate reduction in heated rocks associated with burning lignite by members of the thermophilic genus *Desulfofundulus*. Using radioactive tracer, metagenomic analysis and cultivation, we found that thermophilic *Desulfofundulus* spores can germinate into vegetative cells at temperatures below 25°C and thus support sulfate reduction also under mesophilic conditions.

## 2. Materials and methods

### 2.1. Study site, sampling, and physicochemical parameters measurements

The geographical location of the Chagan-Uzun lignite open pit in the Altai Mountains was described earlier (Kadnikov et al., 2018). We regularly sampled heated rocks with visible signs of underground coal smoldering in period between 2015 and 2021 years. The AL36 sample used to isolate the pure culture was collected on August 08, 2019. The heated rock temperature was measured by inserting an electrode into the rock/soil with a pH-meter HI 8314 (Hanna Instruments, Vöhringen, Germany). On-site measurements of CO, H_2_S, CH_4_, and H_2_ were carried out using a portable gas analyser OKA-T (Informanalitika, Russia) with an electrochemical sensor with a relative error of 25% vol. Sulfate concentrations were measured in heated rock or soil extracts prepared as follows: 2.5 g of rock/soil sample was dissolved in 25 mL of deionized water and shaken on a reciprocating shaker at 75 rpm for 1 h. An aliquot of the rock/soil extract was filtered through a 0.22 μm filter and used for ion chromatography measurements (Dionex, CA, United States). The mineralogical composition of minerals associated with lignite burning was characterised by X-ray diffraction (XRD) using a Shimadzu XRD-6000 diffractometer, as described previously (Ikkert et al., 2013).

### 2.2. Measurement of sulfate reduction rate with a radioactive tracer

Radioactive sulfate was used to determine the sulfate reduction rate (SRR). Samples of heated rock or soil were placed into sterile 5 mL syringes closed with butyl rubber stoppers, which received aliquots (200 μL) of Na_2_^35^SO_4_ (4 μCi “Perkin-Elmer”, United States) by injection through the butyl rubber stopper. The syringes were incubated in the dark at two temperatures, 60°C and 23°C, for 24 h, followed by the addition of 1 mL of 2 M KOH to stop microbial activity and fix sulfide. Subsequent analytical procedures to measure reduced ^35^S species formed in heated rock/soil samples were carried out in the laboratory. Radioactivity was measured in the acid volatile sulfide (AVS) fraction, which included H_2_S and FeS, and the chromium-reducible sulfur (CRS) fraction, which included pyrite, elemental and organic sulfur, as described previously (Karnachuk et al., 2005, 2023). Briefly, in the first step, reduced sulfur in the form of monosulfides, including FeS (AVS), was separated by acid distillation from other forms of reduced sulfur (CRS) and unreacted sulfate. The radioactivity of the AVS fraction and unreacted sulfate was determined independently. In the second stage, acid distillation in the presence of Cr^2+^ reduced pyrite (FeS_2_), elemental sulfur and organic sulfur to H_2_S. The radioactivity of this fraction of reduced sulfur species was determined as CRS. The effluent gas stream from the acid distillation in both stages was bubbled into traps filled with a scintillation cocktail containing 2-phenylethylamine. The reagents and conditions for acid distillation have been described previously (Karnachuk et al., 2005, 2023). Radioactivity was measured on a TRI-Carb TR 2400 (Packard, United States) liquid scintillation analyzer. The rate of sulfate reduction, v in nmol S cm^−3^ day^−1^, was calculated as.


$$v=H^{35}S^{-}\;{{\mathrm{SO}}_{4}}^{2-}/\left(H^{35}S^{-}{+}^{35}{{\mathrm{SO}}_{4}}^{2-}\right)\;t$$

where H^35^S^−^ is the concentration of radioactive, reduced sulfur (nmol cm^−3^); ^35^SO_4_^2−^ is the concentration of radioactive unreduced sulfate (nmol cm^−3^); SO_4_^2−^ is the *in situ* concentration of sulfate; and t is the incubation time (days). The mean SRR and standard deviation were calculated from triplicate incubations.

### 2.3. SRB enrichment, pure culture isolation and characterization

An initial enrichment from AL36 heated rock sample was prepared in modified Widdel and Bak (WB) freshwater medium (Widdel and Bak, 1992) that contained (per liter) 0.15 g Na_2_SO_4_, 0.2 g KH_2_PO_4_, 0.25 g NH_4_Cl, 1 g NaCl, 0.4 g MgCl_2_·6H_2_O, 0.5 g KCl, 0.113 g CaCl_2_, 2 mL of vitamin solution, 1 mL of microelement solution, 1 mL each of Na_2_SeO_3_ (final concentration 23.6 μM) and Na_2_WO_4_ (24.2 μM) solutions (Widdel and Bak, 1992). The medium was adjusted to pH 7.2 with NaHCO_3_ solution pH, and 0.2 mL of Na_2_S·9H_2_O stock solution was used as a reducing agent. Each cultivation vial received a Fe^0^ wire as previously described (Karnachuk et al., 2019). Lactate (18 mM) and formate (7.5 mM) were used as electron donors for initial enrichment. The enrichments were incubated at 60°С. A pure culture was isolated by repeated serial dilutions and culture exposure at 90°C for 20 min. The *16S rRNA* gene was amplified using the primer pair 27F and 1492R (Lane, 1991) and sequenced commercially by Syntol Co. (Moscow, Russia) using the Sanger method.

Growth of the isolated pure culture was analyzed with the following electron donors: 7.5 mM formate, 9 mM acetate, 13.5 mM propionate, 7 mM butyrate, 7 mM pyruvate, 4.5 mM succinate, 9 mM fumarate, 7.5 mM malate, 5 mM fructose, 5 mM glucose, 3 mM sucrose, 25 mM ethanol, 11 mM glycerol, and 1 g L^−1^ peptone (all Sigma-Aldrich). Carbohydrate stock solutions were sterilised using polyethersulfone 0.22 μm Millex-GP filter units (Merck Millipore, Darmstadt). If growth was observed, the culture was subcultured at least five times in the presence of each electron donor to confirm their utilization.

For experiments with spores, strain Al36 was grown with lactate in a liquid medium in sealed, headspace-free 500 mL serum bottles at 65°С. To remove the iron precipitate formed in the sulfidogenic culture, the culture was centrifugated at 1,000 × g for 2 min before cell harvesting. Bacteria from a 2.5 L culture in stationary phase (120 h) were harvested by centrifugation at 11,000 x g for 40 min and washed with 1× PBS buffer. The cells were additionally washed with 1 N HCl in order to remove iron sulfide precipitated on the spores. Vegetative cells were killed by exposing the culture at 95°C for 1.5 h and rinsing with 1× PBS buffer. The spores were suspended in fresh medium with CO (5%) as the sole electron donor and incubated at 8°C, 15°C, and 20°C. Growth was determined by microscopic cell counts using an Axio Imager A1 microscope in triplicate samples. Specific growth rate was calculated from the cell counts during the exponential phase of growth. H_2_S was measured colorimetrically with the methylene blue method (Cline, 1969) in triplicate using a Smart Spec Plus spectrophotometer (Bio-Rad Laboratories, Hercules, CA).

Cell morphology and germinating spores were observed by phase contrast microscopy using an Axio Imager A1 microscope and by transmission electron microscopy (TEM) of ultrathin sections, as previously described (Bukhtiyarova et al., 2019). Briefly, for transmission electron microscopy, germinating spores from 500 mL culture bottles incubated at 20°С for 27, 144 and 192 h were harvested by centrifugation at 11,000 × g for 40 min and washed with 1× PBS buffer. Germinated spores incubated at 15°С were harvested for TEM after 118 h by centrifugation under the same conditions. Fixation of pelletized samples in glutaraldehyde, staining with osmium tetroxide, and dehydration with ethanol have been described previously (Ikkert et al., 2013). Ultrathin sections (60–100 nm) were prepared with an ultramictome (Ultratome III, LKB, Stockholm, Sweden) and viewed with a JEM-100 CXII electron microscope (JEOL, Tokyo, Japan) at a voltage of 80 kV.

### 2.4. *16S rRNA*-based microbial community profiling

PCR amplification of 16S ribosomal RNA gene fragments spanning the V3–V4 variable regions was carried out using the universal primers 341F (5′-CCTAYGGGDBGCWSCAG-3′) and 806R (5′-GGACTACNVGGGTHTCTAAT-3′) (Frey et al., 2016; Wasimuddin et al., 2020). PCR fragments were barcoded using the Nextera XT Index Kit v.2 (Illumina, USA) and sequenced on the Illumina MiSeq (2 × 300 nt paired-end reads). Paired-end overlapping reads were merged using FLASH v.1.2.11 (Magoč and Salzberg, 2011). Low-quality reads were filtered, and the remaining sequences were clustered into operational taxonomic units (OTUs) at 97% identity using the Usearch program (Edgar, 2010). Chimeric sequences and singletons were removed during clustering by the Usearch algorithm. To calculate OTU abundances, all reads were mapped to OTU sequences at 97% global identity threshold by Usearch. OTUs composed of only a single read were discarded. The taxonomic identification of OTUs was performed by searches against the SILVA v.138rRNA sequence database using the VSEARCH sintax algorithm (Rognes et al., 2016).

### 2.5. Strain Al36 genome sequencing

The Al36 strain was grown in modified WB medium with lactate (18 mM) as electron donor for genomic DNA isolation. Genomic DNA was isolated from strain Al36 using a DNeasy Power Soil DNA isolation kit (Mo Bio Laboratories, Carlsbad, CA, United States). The shotgun genome library was prepared using the NEBNext Ultra II DNA library prep kit (New England Biolabs, Ipswich, MA, United States). The sequencing of this library on an Illumina MiSeq instrument in a paired reads mode (2 × 300 nt) generated 845,338 read pairs. Low quality sequences were trimmed using Sickle v.1.33 (*q* = 30).^1^ Trimmed reads were merged with FLASHv.1.2.11 (Magoč and Salzberg, 2011). Resulting reads were *de novo* assembled using SPAdes v. 3.15.4 (Bankevich et al., 2012) into 101 contigs longer than 200 bp.

Gene search and annotation were performed using the RAST server 2.0 (Brettin et al., 2015) followed by manual correction of the annotation by comparing the predicted protein sequences with the National Center for Biotechnology Information (NCBI) databases. The presence of specific conserved domains was verified for some genes related to sulfate reduction and utilization of organic substrates. The search and classification of hydrogenases was carried out using HydDB tool (Søndergaard et al., 2016).

Digital DNA:DNA hybridization calculation was performed using the Genome-to-Genome Distance Calculator (GGDC) online platform.^2^ The average nucleotide identity (ANI) values were calculated using ANI calculator from the Enveomics Collection (Rodriguez-R and Konstantinidis, 2016).

### 2.6. Sequencing of metagenomic DNA and identification of *dsrABD* gene sequences

The metagenomic DNA of sample AL36 was sequenced using the Illumina HiSeq2500 platform according to the manufacturer’s instructions (Illumina). The sequencing of a paired-end (2 × 150 nt) TruSeq DNA library generated 15,336,062 read pairs. Open reading frames (ORFs) with a minimum length of 96 nucleotides were predicted in all Illumina reads using OrfM v.0.7.1 (Woodcroft et al., 2016). HMM profiles for *dsrA* and *dsrB* from TIGRFAM (Haft et al., 2003), and for *dsrD* from Pfam (Finn et al., 2014) were searched against all predicted ORFs using rpsblastv.2.12.0 with 1e^−3^ *E*-value cutoff. ORFs that had significant identity to the HMM profiles were further searched against UniRef100 database using diamond v.0.9 (Buchfink et al., 2015) with1e^−3^ *E*-value cutoff. ORFs that had non-*dsr* genes among top 10 UniRef100 hits were excluded. The NCBI taxonomy for every ORF homolog from UniRef100 database was identified using NCBI-taxonomist v.1.2.1 program.^3^

## 3. Results

### 3.1. Physicochemical characteristics of the sampling site

**Figure 1:**
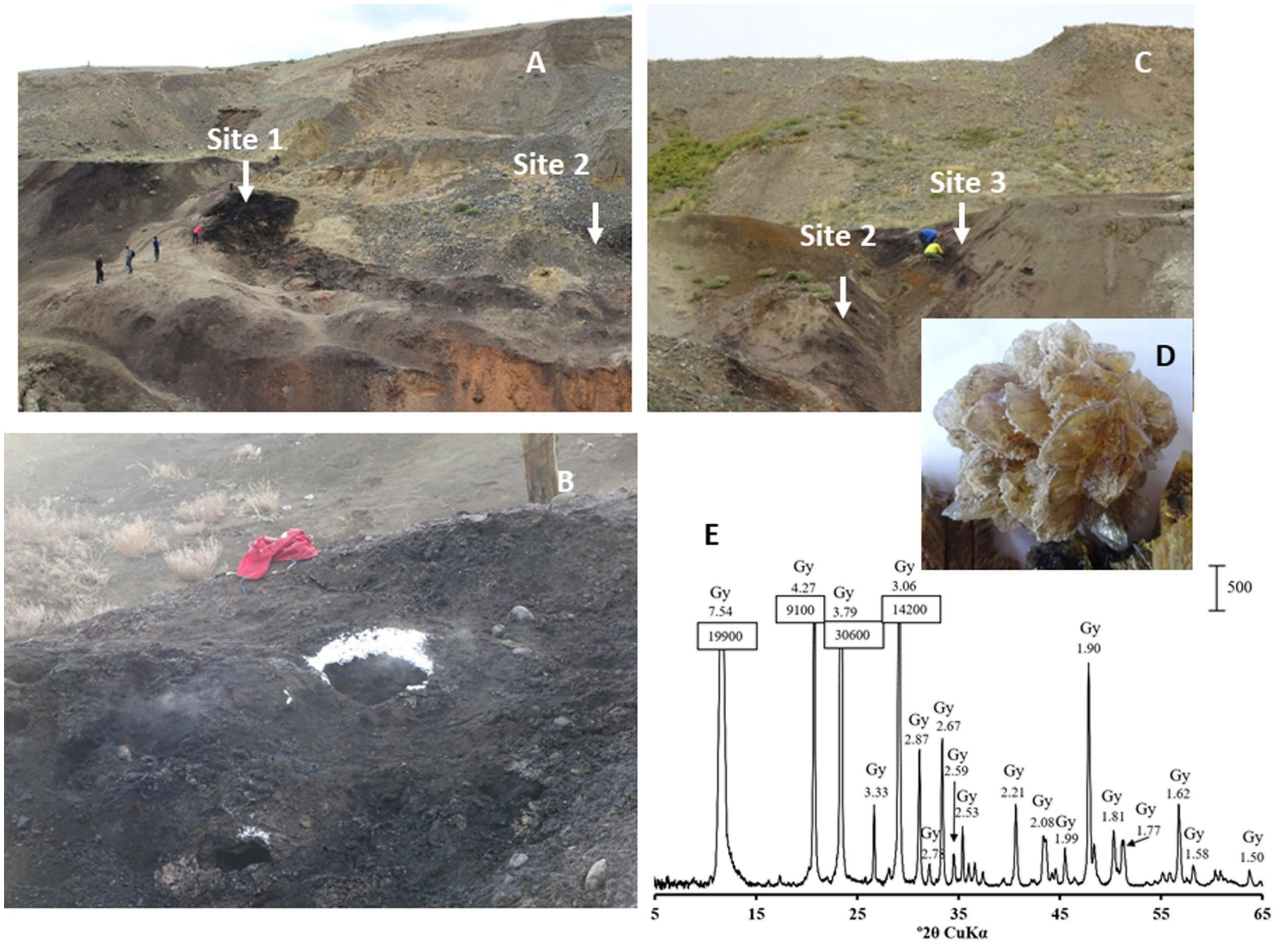
**(A)** Lignite strip mining open pit **(B)** Sampling site 1. Note the ice crystals precipitated around the vent due to the ambient air temperature of −15°C on November, 19th, in the time of collection AL61 and AL62 samples. **(C)** Sampling site 2 and background control sampling site 3 in the same lignite open pit without traces of smouldering lignite. **(D)** Rose gypsum crystal (CaSO_4_) and **(E)** its diffractogram showing gypsum as the only crystalline phase of the crystal.

The Taldy-Dyurgunskoye coalfield is located in the western part of the Chuya valley, 5 km to the southwest of the Chagan-Uzun village and the famous Chuysky Tract connecting Siberia with Mongolia and China (50°4′31.54″N, 88°18′47.4″E). Brown coal seams at an elevation of 1910–1770 meters above sea level began to be mined by open pit method in the late 1980s. Mining was halted due to poor coal quality and spontaneous combustion. We regularly sampled the open pit with visible signs of underground coal smouldering (Figure 1) from 2015 to 2021. The study targeted three sites in the open pit: a mixture of heated rocks and lignite near the vent (fumarole) emitting smoke (site 1) (Figures 1A,B); heated rocks adjacent to a semi-solid bitumen-like outcrop (site 2) (Figures 1A,C); soil of the low temperature background control site without signs of underground coal burning (site 3) (Figure 1C).

The physicochemical characteristics of the sampling sites are shown in Table 1. The average H_2_S concentration at site 1 varied from 8.5 to 60.0 mg m^−3^. The CO concentration was measured during three sampling sessions and comprised 1.0 mg m^−3^ in a 10–15 cm layer and increased with depth up to 3.0 mg m^−3^ in August 2021. In November 2021, CO was not detected. A characteristic feature of heated rocks associated with smoldering lignite is constant temperature fluctuations due to the intensity of heat steam from underground, ambient air temperature, wind, humidity and precipitation. Most of the samples were collected at a depth of 10–15 cm below the heated rock surface to provide isolation from the surrounding atmosphere. The temperature at sampling site 1 at a depth of 10–15 cm was 34.9°C in November and 68.8°C in August. The temperature at a depth of 80 cm in August 2021 was 70°C.

**Table 1:** Characteristics of the sites at the time of sampling.

Sample	Date of sampling	Depth, cm	*T*	pH	H_2_S, mg m^−3^	CO, mg m^−3^	CO_2_, mg m^−3^	CH_4_, mg m^−3^
**Site 1 underground coal fire and vents field**								
AL36	04.08.2019	10–15	68.0	nd	nd	nd	nd	nd
AL51	12.08.2020	10–15	68.8	7.42	60.0	nd	nd	nd
AL54	10.08.2021	10–15	55.0	nd	8.5	1.0	0.47	0.2
AL56	10.08.2021	80	70.0	nd	10.7	3.0	1.4	0
AL61	19.11.2021	40	50.0	nd	nd	nd	nd	nd
AL62	19.11.2021	10–15	34.9	nd	7.7	0	0.52	0.36
**Site 2 bitumen discharge field**								
AL37	04.08.2019	10–15	78.0	nd	nd	nd	nd	nd
AL52	12.08.2020	10–15	70.1	7.4	nd	nd	nd	nd
**Site 3 background control soil, no burning**								
AL58	10.08.2021	10–15	23.0	nd	nd	nd	nd	nd

nd, not determined.

X-ray diffraction patterns (XRD) showed the presence of gypsum (CaSO_4_), elemental sulfur (S), and chalcopyrite (CuFeS_2_) in the heated rock. Evaporate rose gypsum (CaSO_4_) crystals were observed all around the smoldering areas of the open pit (Figures 1D,E). Aluminium sulfate, alunogen, Al_2_(SO_4_)_3_17H_2_O, was also detected in most of the samples.

### 3.2. Sulfate reduction rate

**Figure 2:**
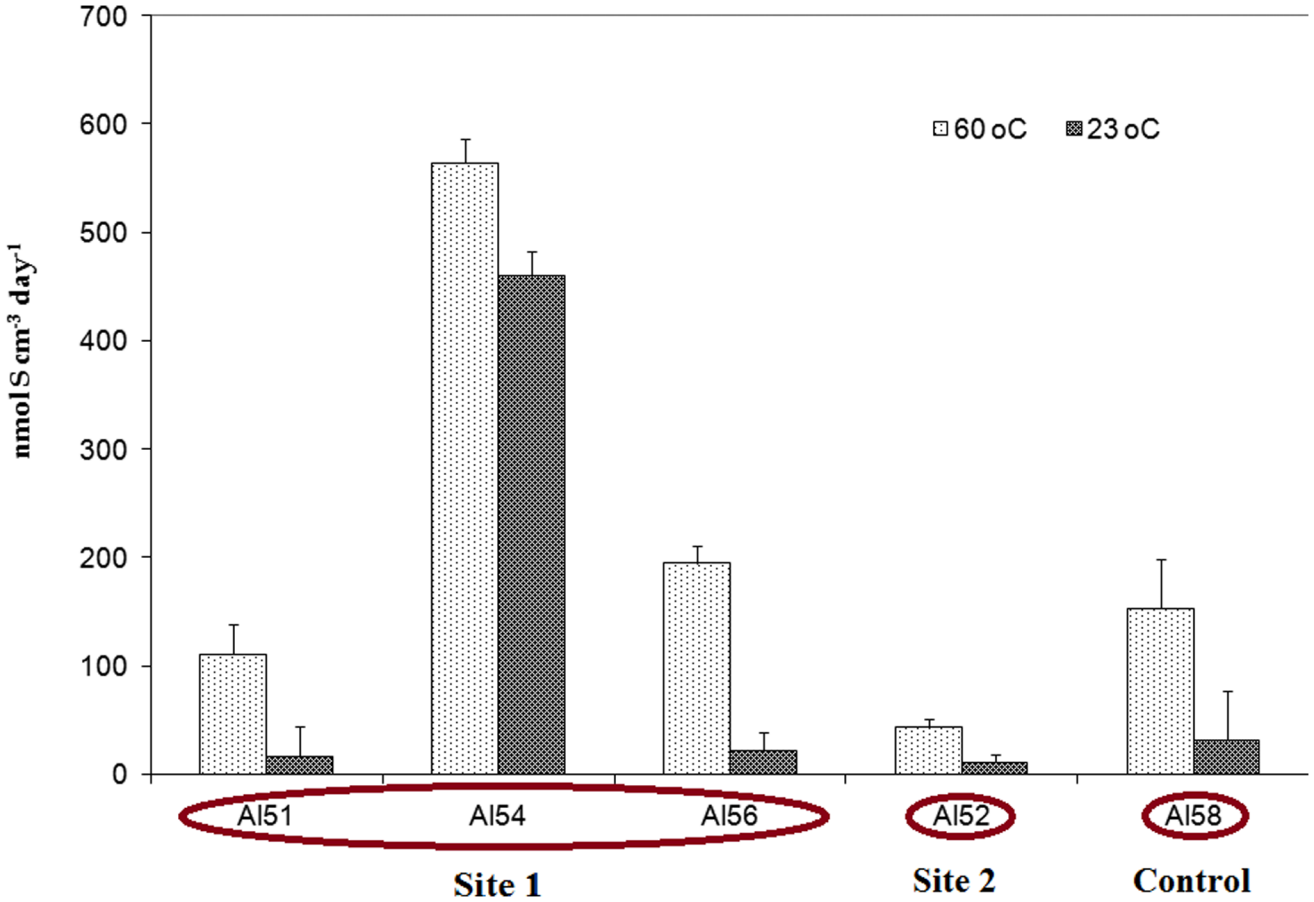
Sulfate reduction rate (SRR) expressed as the total amount of sulfur in the AVS and CRS fractions, measured in experiments with ^35^SO_4_^2−^ tracer in heated rock and soil samples from sites 1, 2 and 3 at an incubation temperature of 60°C and 23°C. The vertical bars show the standard deviation.

The sulfate reduction rate (SRR) measured with a radioactive sulfate tracer was extremely high and varied from 43.9 ± 11.9 (site 2) to 564 ± 21.9 nmol S cm^−3^ day^−1^ (site 1) (Figure 2). For the SRR measurement the heated rock and soil samples were withdrawn with syringes and incubated in a thermostat at a temperature of 60°C, a value close to the average ambient conditions of the biotope. In addition, the parallel syringes were incubated at room temperature 23°C. Surprisingly, the SRR measured at 23°C was slightly lower than at 60°C, but comprised values of the same order of magnitude as the thermal samples (Figure 2). This fact implies that there is a mesophilic microbial community in the thermal biotope that can reduce sulfates at a high rate at lower temperature, which can be beneficial for biotopes characterised by highly variable temperature. SRR measurements at the control site 3 with no burning and at an ambient air temperature of 23°C at the time of sampling (sample AL58) demonstrated that the community was able to reduce sulfate at a high rate of 154 ± 45 nmol S cm^−3^ day^−1^ at 60°C, and 32.3 nmol S cm^−3^ day^−1^ at 23°C.

### 3.3. Microbial community composition

**Figure 3:**
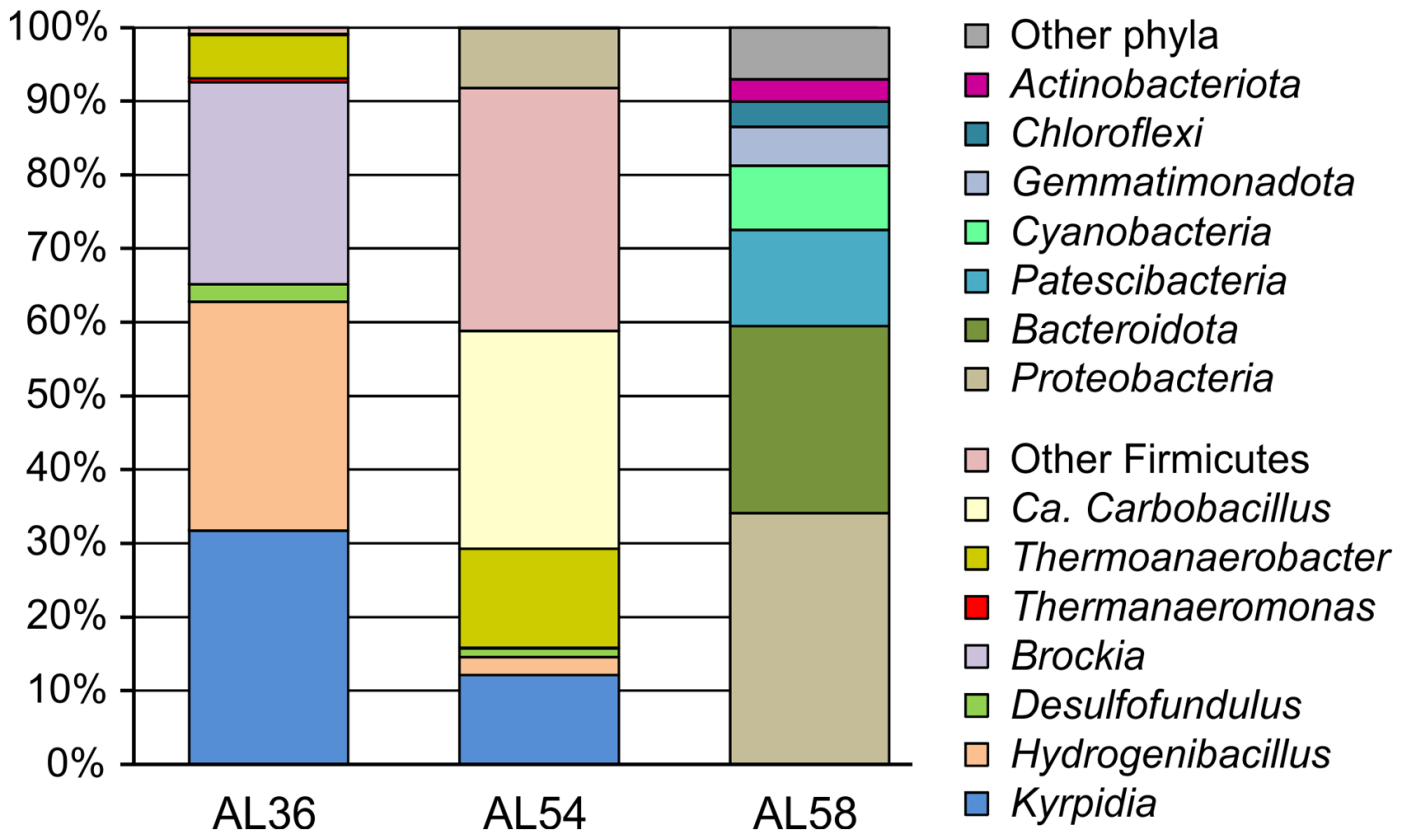
The relative abundance of taxonomic groups of microorganisms according to *16S rRNA* gene profiling in heated rock samples AL54 and AL36 (site 1) and the control site without burning AL58 (site 3). *Firmicutes* accounted for only 0.05% of the *16S rRNA* gene sequences in AL58 sample from the control site 3 (not shown).

Composition of microbial communities of heated rock samples AL36 and AL54 (both site 1), as well as control soil AL58 (site 3) was characterized by *16S rRNA* gene profiling. The pool of reads retrieved from the coal burning site 1 was dominated by *16S rRNA* gene sequences of bacterial origin, and archaeal *16S rRNA* gene reads were revealed only in samples AL54 (site 1) and AL58 (site 3) and accounted for less than 0.04% of the total. Most of the community in two heated rock samples was represented by the phylum *Firmicutes* whose share was almost 100% in AL36 (site 1) sample and about 92% in the sample AL54 (site 1) (Figure 3). The microbiome of sample AL36 (site 1) was dominated by the genera *Kyrpidia* (31.7% of all *16S rRNA* gene sequences), *Hydrogenibacillus* (31.0%), and *Brockia* (27.4%). All the genera comprise thermophilic facultatively chemolitoautotrophic bacteria oxidizing molecular hydrogen under aerobic conditions (*Kyrpidia* and *Hydrogenibacillus*) or anaerobically using elemental sulfur as an electron acceptor (*Brockia*) (Schenk and Aragno, 1979; Bonjour and Aragno, 1984; Klenk et al., 2011; Perevalova et al., 2013). Members of the genus *Thermoanaerobacter*, comprising anaerobes that can grow organoheterotrophically by fermentation or anaerobic respiration with inorganic electron acceptors, in particular sulfur and thiosulfate (Stackebrandt, 2014), accounted for about 5.9% of the community. Potential sulfate reducers were represented by the genera *Desulfofundulus* (2.4%) and *Thermanaeromonas* (0.5%).

Microbial community of the sample, AL54 (site 1), collected from the same site 1 in 2 years after sampling AL36 (site 1), was dominated by *Candidatus* Carbobacillus (29.6%), facultatively anaerobic heterotrophs that can use molecular hydrogen as an energy source (Kadnikov et al., 2018). However, most of *Firmicutes* (33% altogether) belonged to uncultured lineages phylogenetically distant form cultured groups and could not be classified even at the order level. Chemolitoautotrophic hydrogenotrophs of the genera *Kyrpidia* (12.1%), *Hydrogenibacillus* (2.5%), and *Brockia* (0.1%) were found in lower relative abundances. Members of the *Thermoanaerobacter* accounted for 13.4% of the community; the share of *Desulfofundulus* was 1.15%, while *Thermanaeromonas* was not detected.

The composition of microbiome at the control site without lignite burning, AL58 (site 3), was typical for soils. The most numerous phyla were the *Proteobacteria* (34.1%), *Bacteroidota* (25.4%) and *Patescibacteria* (13.1%), while *Firmicutes* accounted for only 0.05% of *16S rRNA* gene sequences. The Firmicutes were represented by members of the genera *Propionispira* (0.02%), *Sulfobacillus* (0.02%), and *Brockia* (0.01%), among the cultivated representatives of which there are no sulfate reducers. *Desulfofundulus* and *Thermanaeromonas* were not found. Therefore, this sample was not analysed in further details.

### 3.4. Metagenomic analysis revealed possible sulfate reducers

To reveal sulfate-reducing bacteria at the coal burning site, we sequenced the metagenome of the AL36 (site 1) sample and searched for genes *dsrA*, *dsrB* and *dsrD*, key markers for the dissimilatory sulfate reduction pathway (Müller et al., 2015; Rabus et al., 2015; Anantharaman et al., 2018). Of about 31 million metagenomics reads, 103, 151 and 24 were mapped to the *dsrA*, *dsrB* and *dsrD* datasets, respectively (Table 2). Most reads (66 for *dsrA*, 106 for *dsrB* nd all 24 for *dsrD*) were taxonomically assigned to the genus *Desulfofundulus*, comprising well known dissimilatory sulfate reducers (Watanabe et al., 2018). Nearly all other *dsrA* and *dsrB* reads represented the genus *Thermanaeromonas*. Cultured members of this genus, *Thermanaeromonas toyohensis* and *Thermanaeromonas burensis* are strictly anaerobic bacteria capable to reduce thiosulfate, but not sulfate (Mori et al., 2002; Gam et al., 2016). However, thermophilic sulfate-reducing strain *Thermanaeromonas* RL80JIV from a geothermally active underground mine was described as well (Kaksonen et al., 2006).

**Table 2:** Taxonomic assignment of *dsr* reads.

Lineage	*dsrA*		*dsrB*		*dsrD*	
	reads	% of the total	reads	% of the total	reads	% of the total
*Desulfofundulus*	66	64	107	70	28	100
*Thermanaeromonas*	36	35	44	29	0	0
Others	1	1	1	1	0	0
Total	103	100	152	100	28	100

### 3.5. *Desulfofundulus* pure culture isolation and characterization

The heated rock sample AL36 (site 1) was used for pure culture isolation. Considering that *16S rRNA* profiling revealed *Desulfofundulus* as a major phylotype with a known ability to sulfate reduction, lactate and formate were chosen as growth substrates for its cultivation. Initial enrichment was set in WB medium with lactate (18 mM) and formate (7.5 mM). Enrichments were incubated at 60°С. No growth was observed with formate, while lactate-emended enrichments demonstrated active sulfidogenesis. Multiple serial dilutions of the lactate-grown enrichments followed by culture exposure at 90°C for 20 min allowed us to obtain a pure culture isolate, which was designated strain Al36.

Cells of strain Al36 are immotile rods, 2.0–4.0 μm long, and 0.5–0.7 μm wide (Supplementary Figure S1). Spherical, centrally placed endospores swell the cells. Spores were detected already in the exponential growth phase, and almost half of all cells formed spores by stationary growth when the strain was grown with pyruvate as an electron donor. The *16S rRNA* gene sequence of strain Al36 was identical to the *Desulfofundulus* OTU detected in the AL36 (site 1) sample in the *16S rRNA* gene profiling experiment and 99.18% similar to that of *Desulfofundulus thermocisternus* DSM 10259. The sequence similarity was above of 98.7%, the species boundary cutoff, assuming that Al36 is a novel strain of *D. thermocisternus* (Supplementary Figure S1C).

The phylogenetic position of strain Al36 was confirmed by its genome analysis. The average nucleotide identity (ANI) between Al36 genome and *D. thermocisternus* DSM 10259 was 96.58%, a value above the species boundary cutoff of 95% (Konstantinidis and Tiedje, 2005; Jain et al., 2018). Likewise, digital DNA:DNA hybridization analysis estimated a 76% probability that strain Al36 and *D. thermocisternus* DSM 10259 belong to the same species. The second close relative of strain Al36 was *Desulfofundulus australicus* AB33 (DSM 11792), with the ANI value of 97.34%. However, a recent publication states that the nomenclatural type of the species *D. australicus*, strain AB33, is no longer available in two publicly accessible international collections (Tindall, 2019), and thus, the species cannot be considered to have valid status. The ANI between the *D. thermocisternus* and *D. australicus* genomes was 97.43%, indicating that all three strains actually belong to the same species (Supplementary Figure S2).

**Figure 4:**
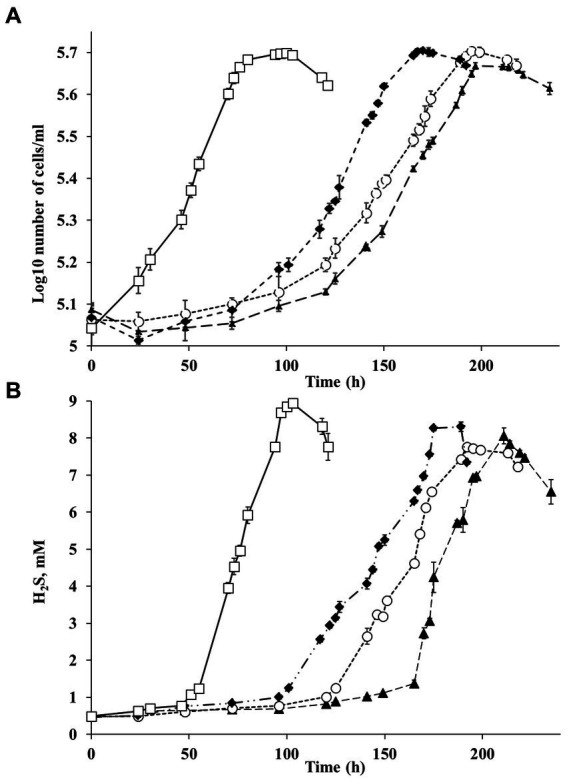
**(A)** Growth of strain Al36 with CO as the sole electron donor and **(B)** H_2_S production during growth with CO at different temperatures: ▲ 50°С (triangles), ○ 55°С (circles), ♦ 60°С (diamonds), □ 65°С (squares). Data are expressed as the means of three replicates, and the vertical bars show standard deviation.

Strain Al36 used a limited number of substrates for sulfate reduction, including CO (5%), lactate, pyruvate, and malate. The strain did not grow with H_2_, formate, acetate, propionate, butyrate, ethanol, glycerol, butanol, fructose, glucose, sucrose, mannose, succinate, fumarate, alanine, cysteine, choline, and peptone as electron donors for sulfate reduction. Strain Al36 was able to grow at temperatures from 50°C to 65°C with an optimum temperature of 65°C, when the specific growth rate reached 0.06 h^−1^ (Figure 4). No growth occurred at 45°C or 70°C.

**Figure 5:**
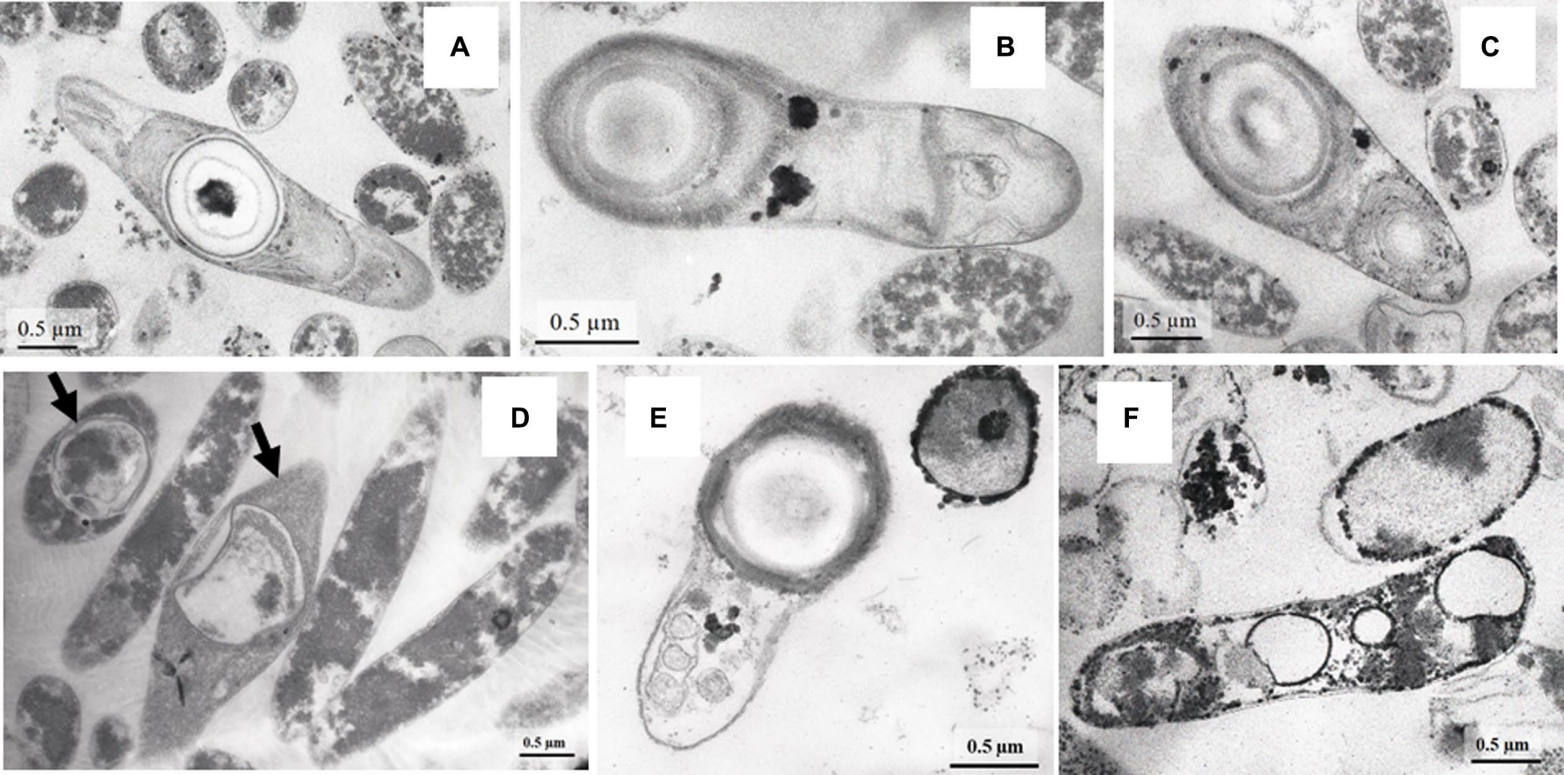
TEM micrograhs of Al36 strain spores germinating in WB fresh medium with CO as the sole electron donor at the “zero time” point at a temperature of 20 **(A–C)** and 15°C **(E)** and cells with “shrunken” cytoplasm, outgrown from Al36 spores after 192 h exposure at 20°C **(D)**. Note the formation of new spores in cells marked with arrows. TEM micrographs revealed electron-dense micro-sized particles of iron sulfide in the periplasm and cytoplasm of cells outgrown from spores of strain Al36 after 118 h exposure at 15°C **(F)**.

**Figure 6:**
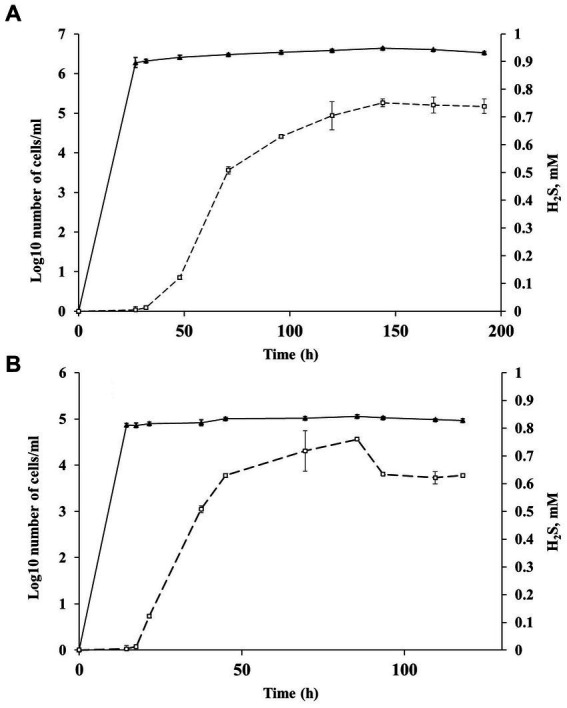
Changes in the number of cells outgrown from spores and sulfide concentration during Al36 spores exposure in fresh WB medium with CO as the sole electron donor at 20 **(A)** and 15°C **(B)**: ▲ −Log10 number of cells/mL (triangles), □ sulfide concentration in the medium (squares). Data are expressed as the mean of three replicates, with the vertical bars indicating standard deviation.

The high SRR measured in samples of heated rocks during their incubation at 23°C and the absence of known mesophilic SRB phylotypes in the *16S rRNA* profiles and metagenome in *in situ* samples raised the question of the agent of the observed process. To answer the question, we carried out a series of experiments with spores of strain Al36. Cells were grown to stationary phase (120 h), when approximately 70% of all cells formed spores. Vegetative cells were killed by exposing the culture at 95°C for 1.5 h. The spores were suspended in fresh medium with CO (5%) as the sole electron donor and incubated at 8°C, 15°C and 20°C. Visual observation showed that spores began to germinate and form vegetative cells as soon as they were placed in a fresh medium even at “zero time” point at a temperature of 15 and 20°C (Figures 5A–C,E). The number of cells outgrown from spores reached 1.9 10^6^ ± 5.1 cell/mL by 27 h of spore culture incubation at 20°C and 7.4 10^4^ ± 0.05 cell/mL by 14.5 h at 15°C (Figure 6). The number of germinated spores during culture incubation at 15°C and 20°C for 192 and 118 h, respectively, did not change significantly. Vegetative cells outgrown from spores at temperatures below the growth range had a “shrunken” damaged cytoplasm and produced new spores (Figure 5D; Supplementary Figure S3). Subcultivation of cells outgrown at 20°C did not succeed, but cells began to divide when transferred to 50°C. Under normal temperature growth conditions, Al36 cells restored the cytoplasm structure.

H_2_S appeared in a culture of germinating Al36 spores after 48 h of incubation at 20°C and increased to 0.738 ± 0.026 mM after 192 h of incubation (Figure 6A). Thus, the outgrown cells were metabolically active and reduced sulfate, but could not divide. H_2_S appeared already after 21.5 h of incubation at 15°C and its concentration in the medium reached 0.629 ± 0.007 mM by 118 h of incubation (Figure 6B). Under this temperature condition, the outgrown vegetative cells precipitated microsized electron-dense particles (Figure 5F). Elemental mapping showed that these electron-dense particles were enriched in S and Fe, which confirms sulfide production by cells outgrowing from spores at 15°C (Supplementary Figure S4). No spore outgrowth was detected at 8°C.

To verify the purity of the Al36 isolate and the absence of other sulfate reducers whose activity at low temperatures could explain the observed production of hydrogen sulfide, we analyzed the data obtained from the sequencing of the culture of strain Al36. In the resulting sequences, 502 reads were found representing the *dsrABD* genes, all of which matched *Desulfofundulus* sp. Al36, which confirms the purity of the Al36 culture. We also analyzed the *16S rRNA* gene from the cells outgrown from spores to confirm the absence of mesophilic sulfate reducer contamination during the subculturing. Sanger sequencing of *16S rRNA* gene fragments amplified with 27F, 907R, and 1,492 primers showed 100% sequence similarity between the *16S rRNA* gene from strain Al36 grown at 50°C and the *16S rRNA* gene from cells outgrown from spores at 20°C (Supplementary Figure S5). Our experiments with spores demonstrate that the thermophilic *Desulfofundulus* sp. Al36 produces spores capable of germinating at temperatures below their normal thermophilic growth range and reduces sulfate to H_2_S over a wide temperature range of 15°C to 65°C.

### 3.6. Genome analysis

The draft genome sequence of *Desulfofundulus* sp. strain Al36 consists of 101 contigs (N50 of 268,081 bp) with a total length of 2,900,077 bp. Mapping of the metagenomic reads of the AL36 sample to the genome of *Desulfofundulus* sp. Al36 showed that the relative abundance of this genotype in the metagenome was 2.1%, which is in good agreement with the 2.4% share of *Desulfofundulus* OTU in the *16S rRNA* gene library.

Genome analysis revealed the presence of a complete set of genes for dissimilatory sulfate reduction, including sulfate adenylyl transferase (*sat*), adenylsulfate reductase (*aprAB*) and dissimilatory sulfite reductase (*dsrABD* and *dsrC*). Genes for the adenylsulfate reductase-associated electron transfer complex QmoABC and sulfite reductase-associated electron transport proteins DsrMK were found as well. Consistently with the capability of strain Al36 to grow autotrophically, its genome contains complete set of genes of the Wood–Ljungdahl pathway for carbon fixation.

The genome encodes monomeric ferredoxin-oxidizing [FeFe] hydrogenase and two multisubunit confurcating [FeFe] hydrogenases. The later enzymes can reversibly couple the evolution of H_2_ to oxidation of reduced ferredoxin and NADH (Greening et al., 2016). All hydrogenases lacked membrane subunits and were predicted to be localized in the cytoplasm. Genome comparisons of strain Al36 with *D. thermocisternus* and *D. australicus* revealed that these strains contain identical sets of hydrogenase genes, that could enable hydrogenotrophic growth. Therefore, the inability of *Desulfofundulus* sp. Al36 to use hydrogen as an electron donor for sulfate reduction was surprising considering that two other strains can grow autotrophically with H_2_ + CO_2_ and sulfate (Nilsen et al., 1996). Moreover, the same types of hydrogenases are encoded in the genome of well characterized hydrogenotrophic sulfate reducer *Desulfofundulus* (former *Desulfotomaculum*) *kuznetsovii* (Visser et al., 2013).

Analysis of the Al36 genome revealed the presence of a single formate dehydrogenase. Besides the catalytic subunit, this enzyme was predicted to comprise the NuoF-like NADH-binding subunit and the electron transfer subunit related to NuoE. Such confurcating enzymes, also encoded in the genomes of *D. thermocisternus*, *D. australicus* and *D. kuznetsovii,* can couple the oxidation of formate to the reduction of ferredoxin and NADH (Visser et al., 2013). Formate degydrogenase was predicted to be localized in the cytoplasm and is probably involved in the carbon fixation via the Wood–Ljungdahl pathway or in the oxidation of formate produced from pyruvate by pyruvate formatelyase in course of fermentative growth.

Genome analysis of *Desulfofundulus* sp. Al36 suggested that the ability of this strain for carboxydotrophic growth coupled to sulfate reduction is associated with the occurrence of four genes encoding the catalytic subunit of anaerobic carbon-monoxide dehydrogenase (CODH). However, none of these genes was clustered with hydrogenase genes. It was previously shown that clustering of CODH and membrane-bound energy-converting hydrogenase genes is a characteristic feature of hydrogenogenic carboxydotrophs such as *Carboxydothermus hydrogenoformans* (Wu et al., 2005) and *Desulfotomaculum carboxydivorans* (Visser et al., 2014). The absence of such cluster is consistent with inability of strain Al36 to grow hydrogenogenically on CO.

The observed growth on lactate is consistent with the presence of lactate permease and two lactate dehydrogenases. Pyruvate and malate could be oxidized in the tricarboxylic acids cycle. However, strain Al36 substantially differed from *D. thermocisternus* and *D. australicus* in the range of substrates supporting growth. *D. thermocisternus* and *D. australicus* can use a broad range of organic acids, alcohols, and carboxylic acids (Nilsen et al., 1996). For instance, both *D. thermocisternus* and *D. australicus* can use ethanol; *D. thermocisternus* grows with propionate, butyrate, propanol, and butanol, while *D. australicus* uses acetate and benzoate. Analysis of the genome of strain Al36 suggested that it has genetic potential for utilization of similar organic substrates. All three genomes contain multiple alcohol dehydrogenase genes, as well as methanol and propionate utilization pathways described in *D. kuznetsovii* (Visser et al., 2013). Genes encoding butyrate kinase, a key enzyme required for the utilization of this substrate, was found in the genomes of Al36 and *D. thermocisternus*, but not in *D. australicus* consistently with inability of the later to grow on butyrate. Perhaps, the observed inability of strain Al36 to utilize hydrogen and above mentioned alcohols and organic acids as electron donors for sulfate reduction could be related to insufficient expression of the relevan t genes under experimental conditions or their non-functionality due to unknown point mutations.

## 4. Discussion

### 4.1. The importance of bacterial sulfate reduction in burning coal seams

Traditionally, marine habitats are considered as biotopes with the most active microbial sulfate reduction due to the high concentration of sulfate, which exceeds all other electron acceptors by more than an order of magnitude (Jørgensen, 1982; Røy et al., 2014; Wasmund et al., 2017). Typical sulfate reduction rates in coastal surface-sediments are in the order of 20 nmol SO_4_^2−^cm^−3^ day^−1^ (Jørgensen, 1982). The average SRR measured by the radiotracer method in marine, organic rich sediments have been reported to be between 5 and 80 nmol SO_4_^2−^ cm^−3^ day^−1^ (Dando et al., 1991, 1995). Microorganisms, including SRB, are highly dependent on temperature, and an increase in temperature generally increases microbial activity. Reported rates of sulfate reduction in marine hydrothermal vents at temperatures between 65 and 100°C in the Mid Atlantic Ridge varied between 122 and 136 nmol SO_4_^2−^ cm^−3^ day^−1^ (Schauer et al., 2011) and at *in situ* temperatures above 100°C SRR was 19–61 nmol SO_4_^2−^ cm^−3^ day^−1^ (Jørgensen et al., 1992). The highest SRR measured in the shallow-sea hydrothermal vent system of Milos at 40°C reached 40 nmol SO_4_^2−^ cm^−3^ day^−1^ (Bayraktarov et al., 2013). Biotopes associated with burning coal seams are characterized by both high sulfate concentrations and elevated temperatures. Sulfates are abundant secondary minerals in burning coalfields (Parafiniuk and Siuda, 2021). Numerous gypsum roses observed on our sampling sites 1 and 2 (Figure 1) are evaporites, where crystal formation occurred as a result of an influx of water containing dissolved calcium sulfate balanced by an outflow of water due to evaporation (Hope et al., 2015). The low-solubility sulfate minerals such as gypsum (CaSO_4_·2H_2_O), barite (BaSO_4_), and anglesite (PbSO_4_) can serve as solid phase electron acceptors for SRB (Karnachuk et al., 2002). Members of the *Desulfobacterota* produced H_2_S from CaSO_4_ with the same rate and quantity as from soluble sulfate. It remains to be elucidated the possibility of using alunogen, Al sulfate, which is often found in burning coal fields (Kadnikov et al., 2021), as a feasible solid-phase electron acceptor for sulfate reduction.

In our study, the SRR measured in heated rock associated with lignite burning seams significantly exceeded the average SRR in marine sediments and varied from 43.9 to 564 nmol S cm^−3^ day^−1^. The high activity of sulfate reducers in organic-rich marine sediments is based on the simultaneous presence of electron donors (low weight organic compounds or dihydrogen) and electron acceptors (sulfate ion). The significant SRR observed in thermal biotopes associated with lignite burning seams is based on non-biological electron donor, CO, originating from coal fires. Emissions from underground burning coal seams can occur not only from vents and cracks on the surface, but also by upward diffusion from rocks and soil (Engle et al., 2011), thus supplying CO to biotopes at a temperature suitable for microbial processes. Thus, the high SRR detected in lignite burning seams is based on the abundance of the electron donor (CO) and acceptor (sulfates) for SRB. Burning coal seams producing CO are ubiquitous throughout the world (Stracher and Taylor, 2004; Kuenzer et al., 2007; Shan et al., 2019), and biogenic H_2_S may represent an overlooked significant flux to the atmosphere.

In addition to smoldering coal, another technogenic source of CO in the environment is a product of incomplete fossil fuels production - syngas (synthetic gas) produced by the chemical industry. An enrichment culture containing *Desulfofundulus*-like SRB utilizing CO and closely related to *D. australicus* was isolated from the syngas (Alves et al., 2020). The authors demonstrated the carboxydotrophic potential of the genus *Desulfofundulus* and its ability to use CO as a cheap electron donor for sulfate reduction in bioremediation processes. The cost and availability of carbon sources and electron donors is the greatest challenge among all the limitations in the application of SRB-based bioremediation processes (Kaksonen et al., 2008).

### 4.2. Low-temperature germinating spores of the thermophilic *Desulfofundulus* contribute to active sulfate reduction under fluctuating temperature conditions

A specific feature of the burning coal seams is constant temperature fluctuations (Duarte et al., 2017). The intensity of heat steam from underground, ambient air temperature, wind, humidity, precipitation and light can all cause temperature changes (Wang et al., 2022). *Desulfofundulus*, which inhabits heated rocks, can anchor in a smoldering coal due to their ability to survive and produce spores efficiently over a wide temperature range from 15°C to 65°C. Uncultivated *Desulfofundulus* were also found in the metagenome of a burning coal site in Gusinoozersk in Transbaikalia, located at a distance of 1,300 km from the Chagan-Uzun lignite site sampled for this study (Kadnikov et al., 2023). The metagenome assembled genome (MAG) of a close relative of *D. australicum*, designated Bu22-1-69, constituted the most abundant genotype (1.1% of the metagenome) among *Firmicutes* with a known ability for sulfate reduction in Gusinoozersk. It is conceivable that *Desulfofundulus* spores can spread over long distances and successfully colonize thermal biotopes where CO and sulfate are available. In terms of the generalists/specialists concept (Bell and Bell, 2020), *Desulfofundulus* is a typical specialist that confines itself to a narrow temperature range of 50°C to 65°C and thus can outperform generalists who tend to adapt to a wider temperature range. On the other hand, being a specialist organism, the Al36 strain possesses a mechanism to disseminate itself at temperatures below its growth range through spore germination and production of new spores.

Despite fluctuations in temperature, *Desulfofundulus* can maintain a significant production of H_2_S through a variety of sulfate respiration modes. Vegetative cells can be active at temperatures from 50°C to 65°C, which is the upper limit for growth, while spores can germinate at low temperatures, forming metabolically active vegetative cells. A significant SRR of 154 ± 45 nmol S cm^−3^ day^−1^ measured in a control soil sample (AL58, site 3) when incubated at 60°C implies that *Desulfofundulus* spores may occur in the soil. The soil at site 3 did not experience burning during the period of our observations of the Chagan-Uzun lignite open pit, but was in close proximity to the gas fumarole in the same open pit and contained secondary sulfates (Figure 1C). According to *16S rRNA* gene profiling, AL58 community was a typical soil microbiome, with the most abundant phyla being the *Proteobacteria*, *Bacteroidota* and *Patescibacteria*, while *Desulfofundulus* were not found. Due to the resilience of bacterial endospores to many lysis buffers and chemicals (Knüpfer et al., 2020), DNA from the spore core may not be properly represented in total DNA extracted from an AL58 soil sample.

Under low-temperature conditions, *Desulfofundulus* spores germinated into persistent vegetative cells that were metabolically active and reduced sulfate but could not divide. The observed phenomenon can be considered as an example of growth-arrest of thermophilic bacterium. The most studied causes of growth arrest in prokaryotes refer to nutrient and energy limitations (Pechter et al., 2017). Typical growth-arrested cells stay viable for several days to years, and are usually able to rapidly resume growth when nutrients become available (Bergkessel et al., 2016), similarly persistent *Desulfofundulus* vegetative cells overgrown at 15 and 20°C resumed growth after being transferred to 50°C in this study. The molecular mechanisms of temperature-triggered spore germination into persistent vegetative cells warrants further research.

The thermophilic spore outgrowth and their metabolic activity at temperatures below the growth minimum may have implications for other spore-forming bacteria of environmental, industrial and clinical relevance. For instance, the presence of spores of the thermophilic *Desulfotomaculum* in the permanently cold marine sediments of Svalbard (Hubert et al., 2009) and Aarhus Bay (de Rezende et al., 2013) was attributed to passive dispersal from an enigmatic hydrothermal source. Endospores detected in cold marine sediments were thought to remain inactive under *in situ* temperature conditions. One of the dominant phylotypes, whose spores were observed in the Aarhus Bay sediment, was a close relative of *D. australicus* (de Rezende et al., 2013) and strain Al36 isolated in this study. It is conceivable that endospores of the thermophilic *Desulfofundulus* can outgrow, produce new spores, and even reduce sulfate to H_2_S at low temperatures in marine sediments, just as was observed in our study of the burning lignite seam.

Apart from deep-sea sediments, thermophiles, mostly spore-forming *Firmicutes*, have been discovered in many cold and temperate systems (DePoy and King, 2023). Our findings of metabolically active thermophilic spores at temperatures below their growth range challenge the notion that many thermophiles can be inactive in mesothermic environments and simply maintained by a continuous input from geothermal sources (Marchant et al., 2008; Zeigler, 2014). It is conceivable that other thermophiles may produce spores capable of outgrowing into metabolizing cells at ambient temperatures below their minimum growth requirements. Further experiments with spores may shed light on the possible involvement of spore outgrowth in the well-known paradox of the ubiquitous thermopile distribution in a predominantly mesothermic world.

## Data availability statement

The datasets presented in this study can be found in online repositories. The names of the repository/repositories and accession number(s) can be found in the article/Supplementary material.

## Author contributions

OK conceptualized the study, prepared, and wrote the original draft. NR wrote the original draft. IP isolated pure cultures, studied their characteristics, and performed experiments. IR and NP measured the *in situ* sulfate reduction rate with a radioactive tracer. VK, AL, IR, NR, and OK collected samples and analyzed physicochemical parameters. MA carried out phylogenetic analysis. VK, AB, and AM sequenced DNA, performed *16S rRNA* gene profiling, metagenomic analysis, and genome analysis. OI performed TEM and XRD analysis. YK and MV performed EFTEM analysis. All authors contributed to the article and approved the submitted version.

## Funding

This study was supported by the Russian Science Foundation Projects 21-14-00114 (to OK, sampling, sulfate reduction rate measurements, pure culture isolation and physiological experiments with pure culture and spores) and 22-14-00178 (to NR, metagenome and genome sequencing and analysis) and the Ministry of Science and Higher Education of the Russian Federation.

## Conflict of interest

The authors declare that the research was conducted in the absence of any commercial or financial relationships that could be construed as a potential conflict of interest.

## Publisher’s note

All claims expressed in this article are solely those of the authors and do not necessarily represent those of their affiliated organizations, or those of the publisher, the editors and the reviewers. Any product that may be evaluated in this article, or claim that may be made by its manufacturer, is not guaranteed or endorsed by the publisher.

## Supplementary material

The Supplementary material for this article can be found online at: https://www.frontiersin.org/articles/10.3389/fmicb.2023.1204102/full#supplementary-material

## Footnotes

^1^ https://github.com/najoshi/sickle

^2^ https://ggdc.dsmz.de/ggdc.php#

^3^ https://pypi.org/project/ncbi-taxonomist/
