# Supplementary material for: The low-temperature germinating spores of the thermophilic Desulfofundulus contribute to an extremely high sulfate reduction in burning coal seams
Source: Front Microbiol. 2023 Sep 15;14:1204102. doi: 10.3389/fmicb.2023.1204102 (PMC10540450; doi:10.3389/fmicb.2023.1204102)
Supplement: Supplementary file 1 [file Data_Sheet_1.docx]

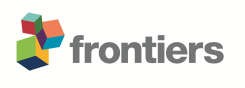


***Supplementary Material***

The low-temperature germinating spores of the thermophilic *Desulfofundulus* contribute to an extremely high sulfate reduction in burning coal seams

**Olga V. Karnachuk ^1*^, Igor I. Rusanov ^2^, Inna A. Panova ^1^, Vitaly V. Kadnikov^3^, Marat R. Avakyan^1^, Olga P. Ikkert^1^, Anastasia P. Lukina^1^, Alexey V. Beletsky^3^, AndreyV. Mardanov^3^, Yuri** **V. Knyazev^4^, Mikhail N. Volochaev**[**^4^, Nikolai V. Pimenov^2^, Nikolai V. Ravin^3^**](mailto:olga.karnachuk@green.tsu.ru)

*** Correspondence:** Olga V. Karnachuk: [olga.karnachuk@green.tsu.ru](mailto:olga.karnachuk@green.tsu.ru)

**1.1 Supplementary Figures**


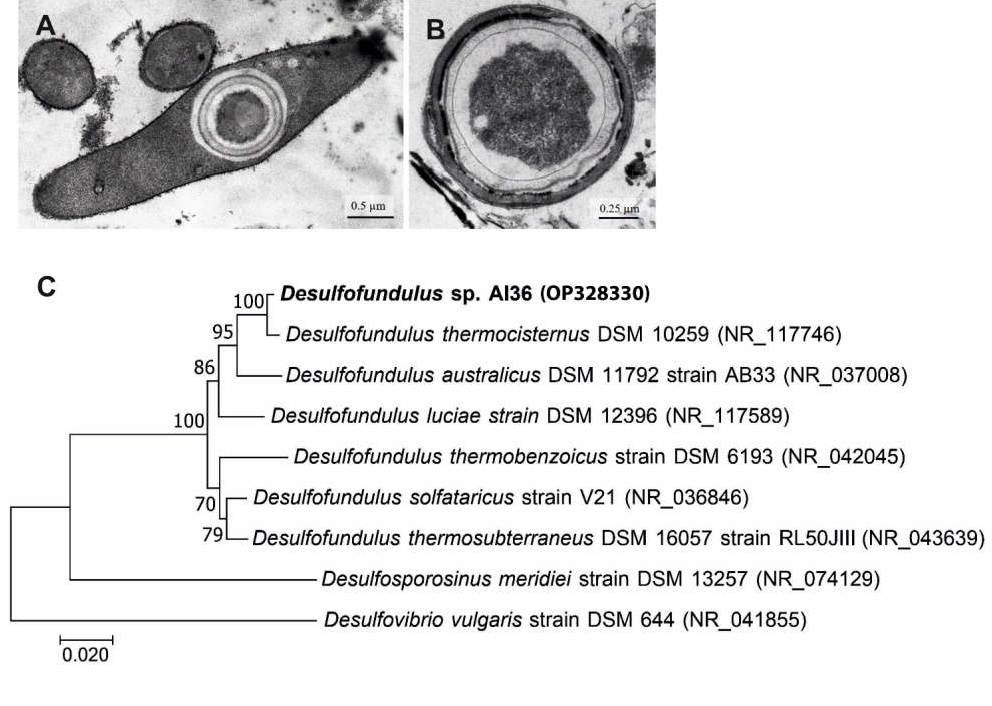


**Supplementary Figure 1. (A)** TEM micrographs of ultrathin layers of strain Al36 showing sporulating cell and (**B**) spore. (**C**)16S rRNA gene-based neighbour-joining tree showing the phylogenetic position of the strain Al36.


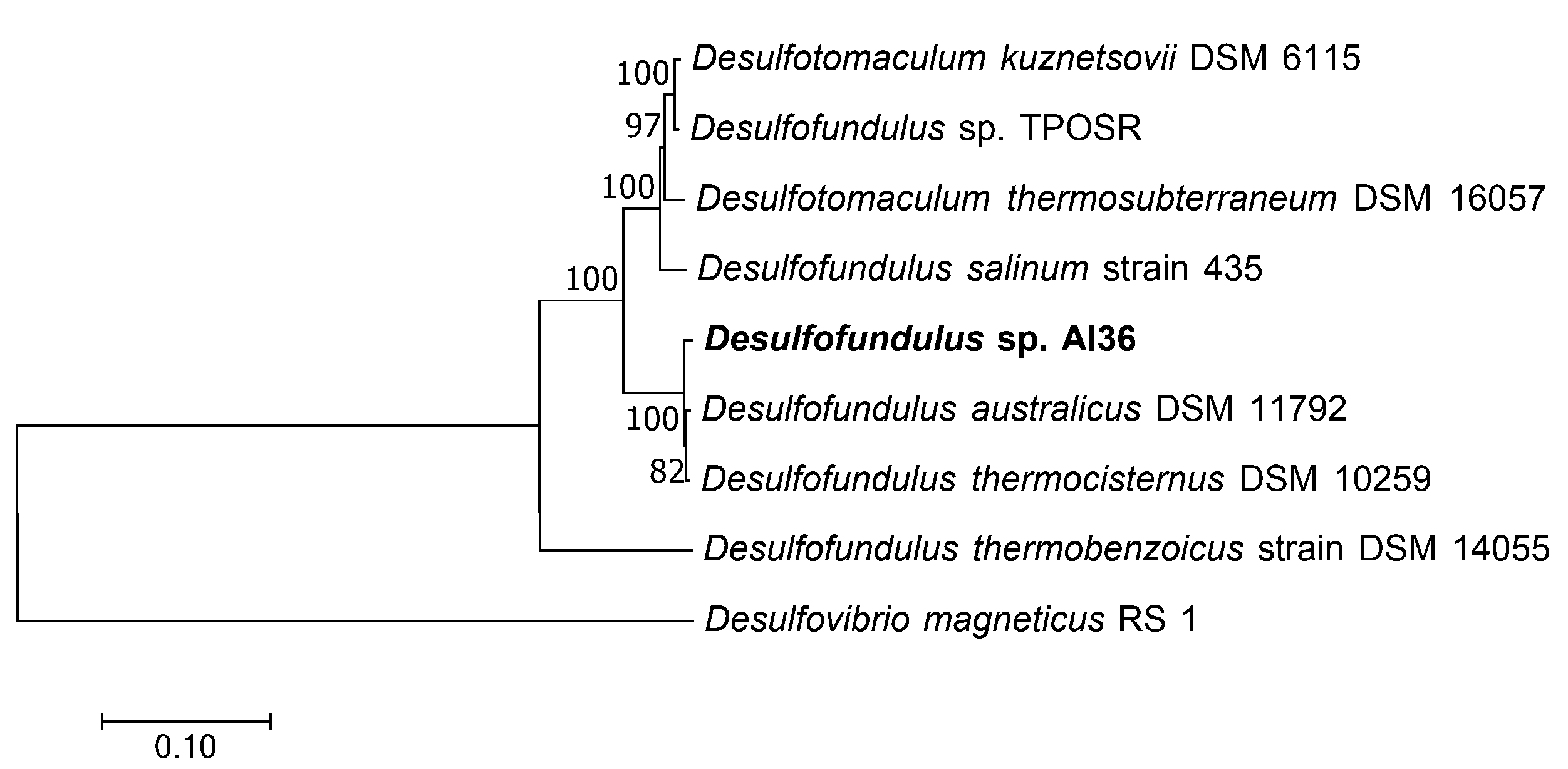


**Supplementary Figure 2.** Neighbour-joining tree of concatenated sequences of 120 bacterial single- copy marker proteins showing the phylogenetic position of the strain Al36.


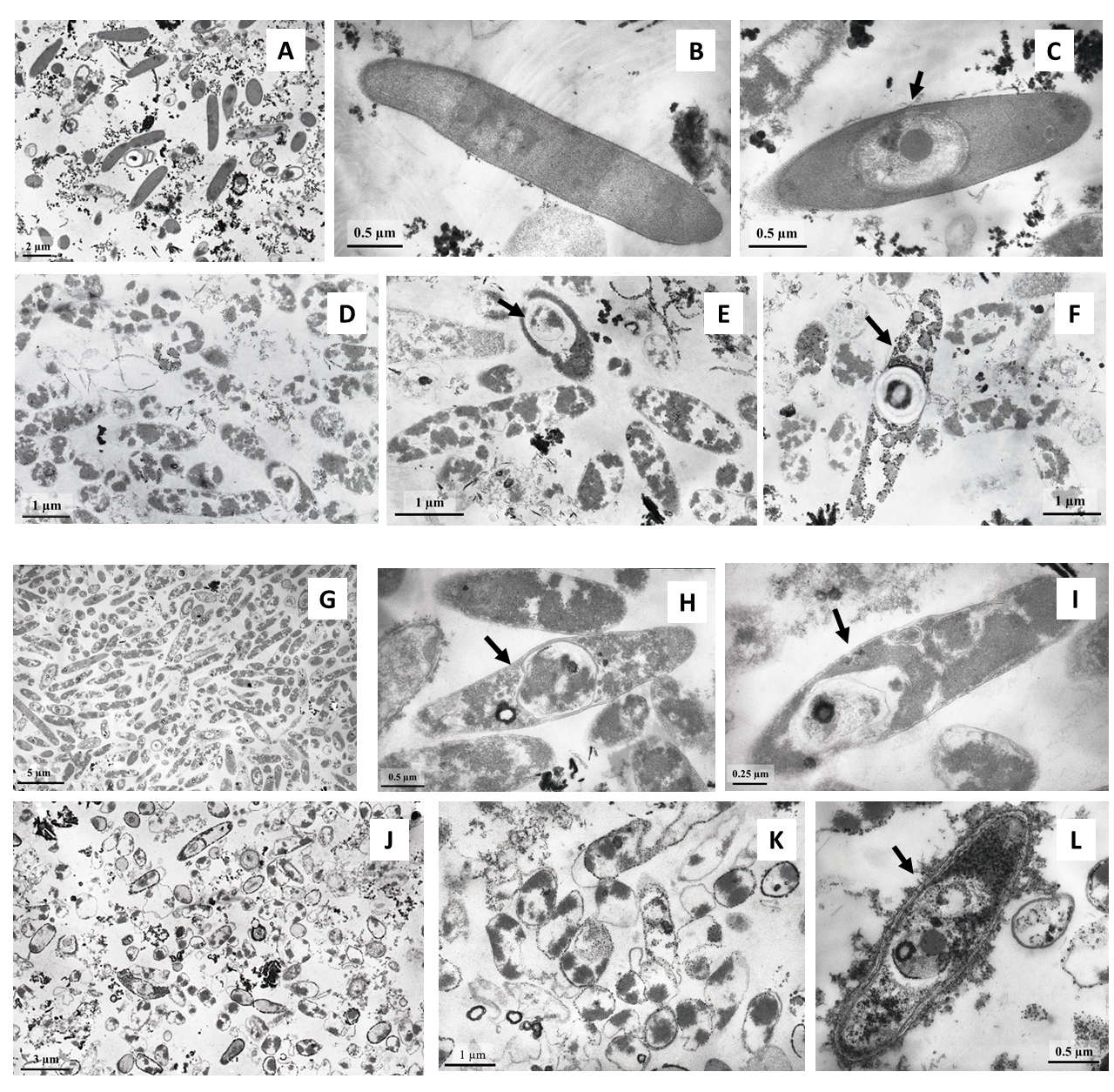


**Supplementary Figure 3.** TEM micrograhs of Al36 strain grown with CO as the sole electron donor for 168 h at 60 °C (**A, B, C**) and cells with “shrunken” cytoplasm, outgrown from Al36 spores after 168 h exposure 20 °C (**D, E, F**); after 216 h exposure at 20 °C (G, H, I); and after 168 h exposure at 15°C (**J, K, L**). Sporulating cells are marked with arrows.


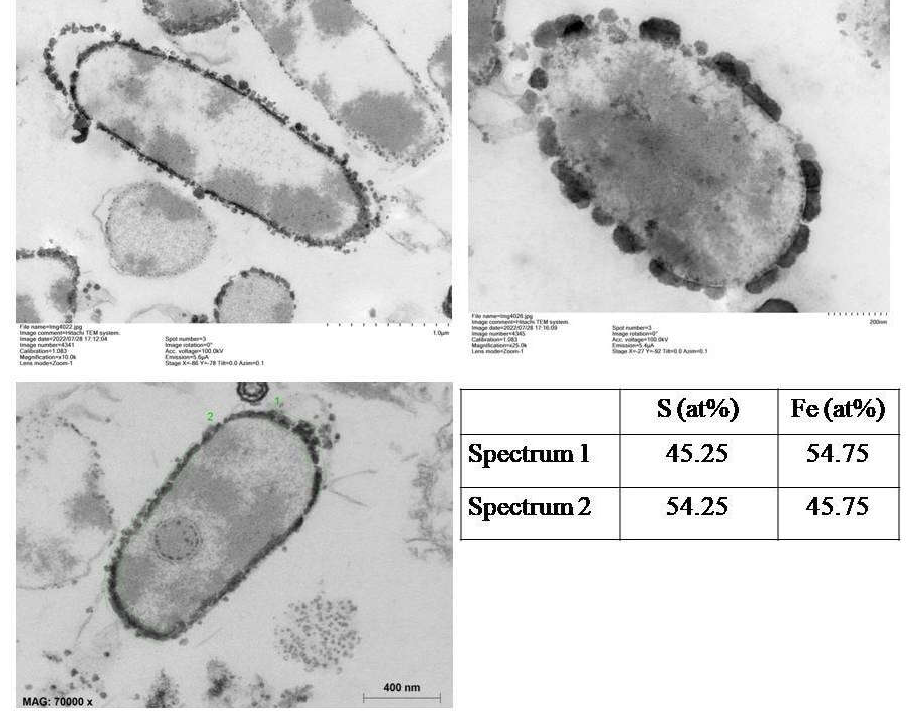


**Supplementary Figure 4.** TEM images of cells outgrown from spores at 15 °C with electron dense particles associated with the cell wall and EFTEM image with the corresponding atomic percentage of S and Fe measured in electron dense particles.


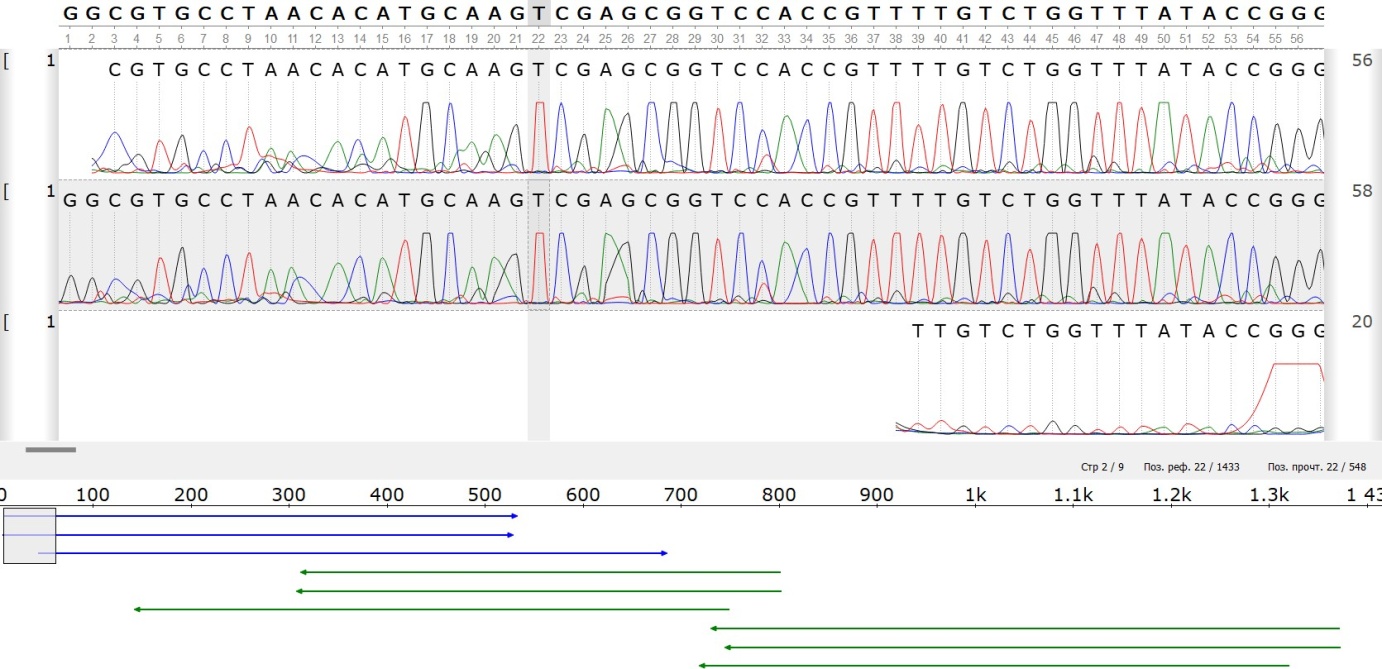
consensus

1 27F

2 27F

3 27F

consensus


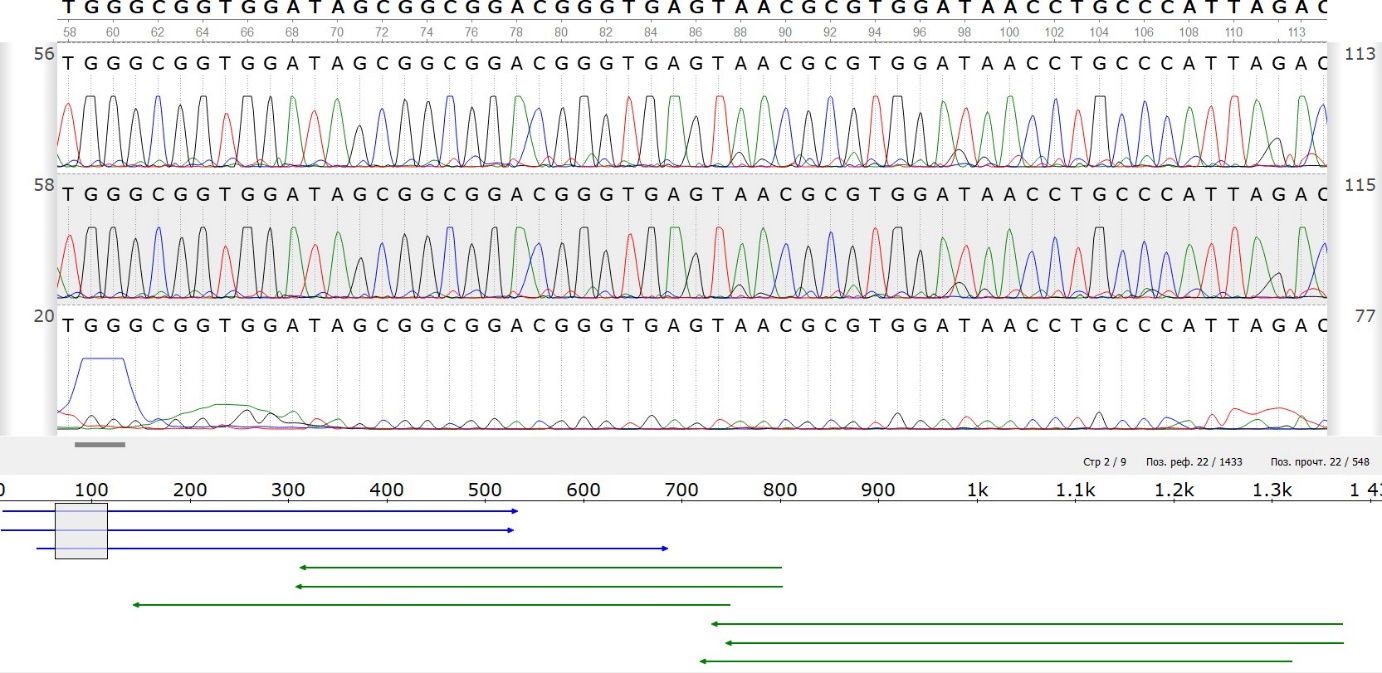

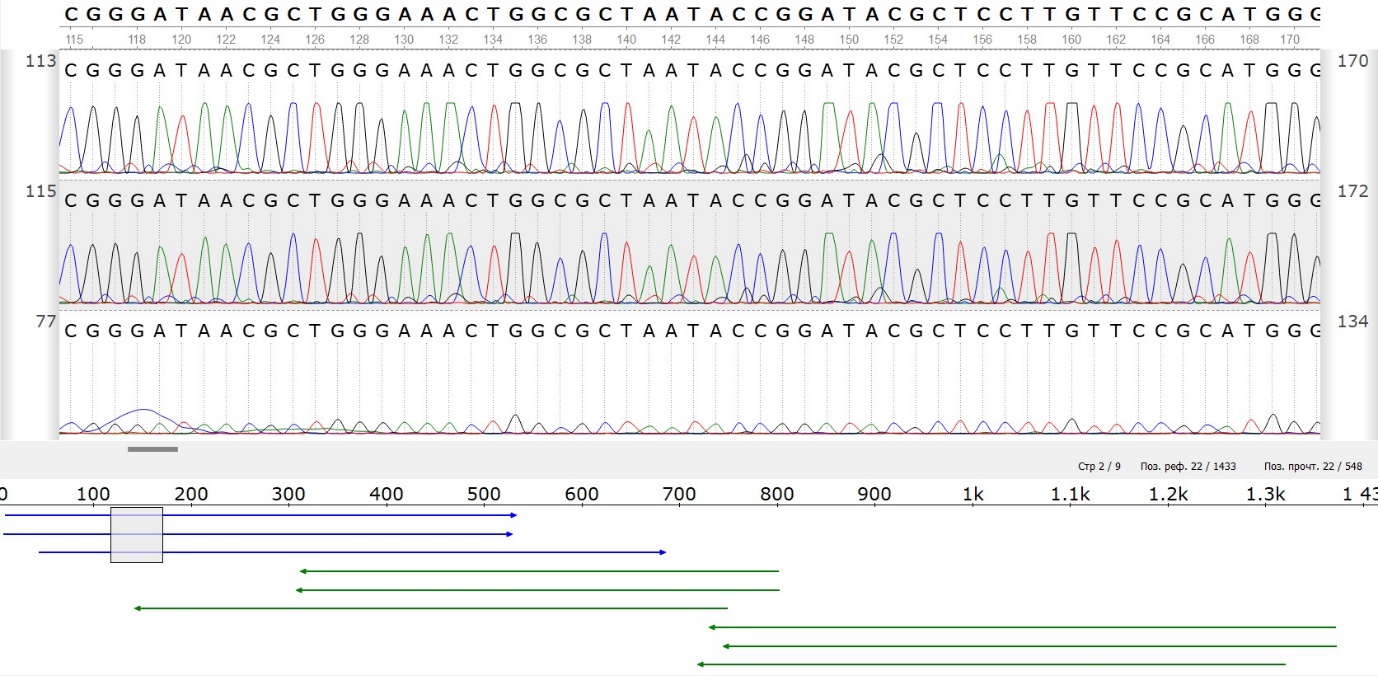


1 27F

2 27F

3 27F

consensus

1 27F

2 27F

3 27F


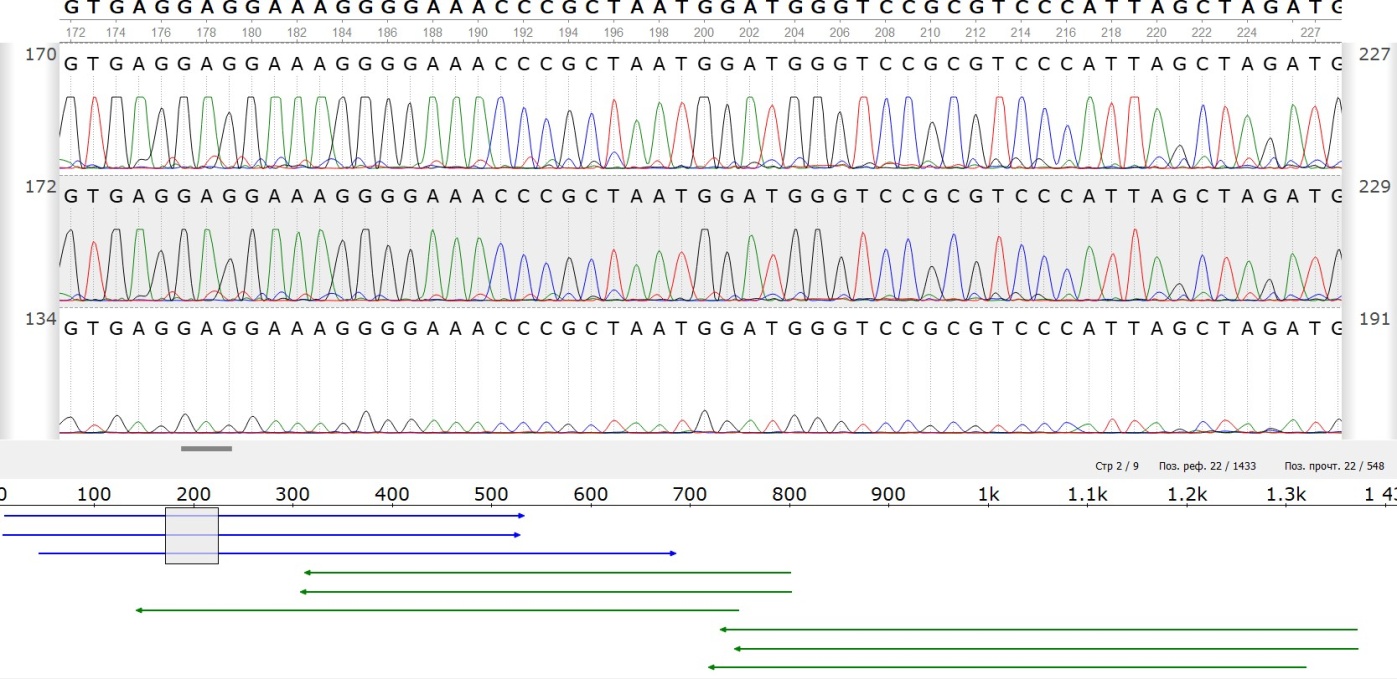
consensus

1 27F

2 27F

3 27F

consensus


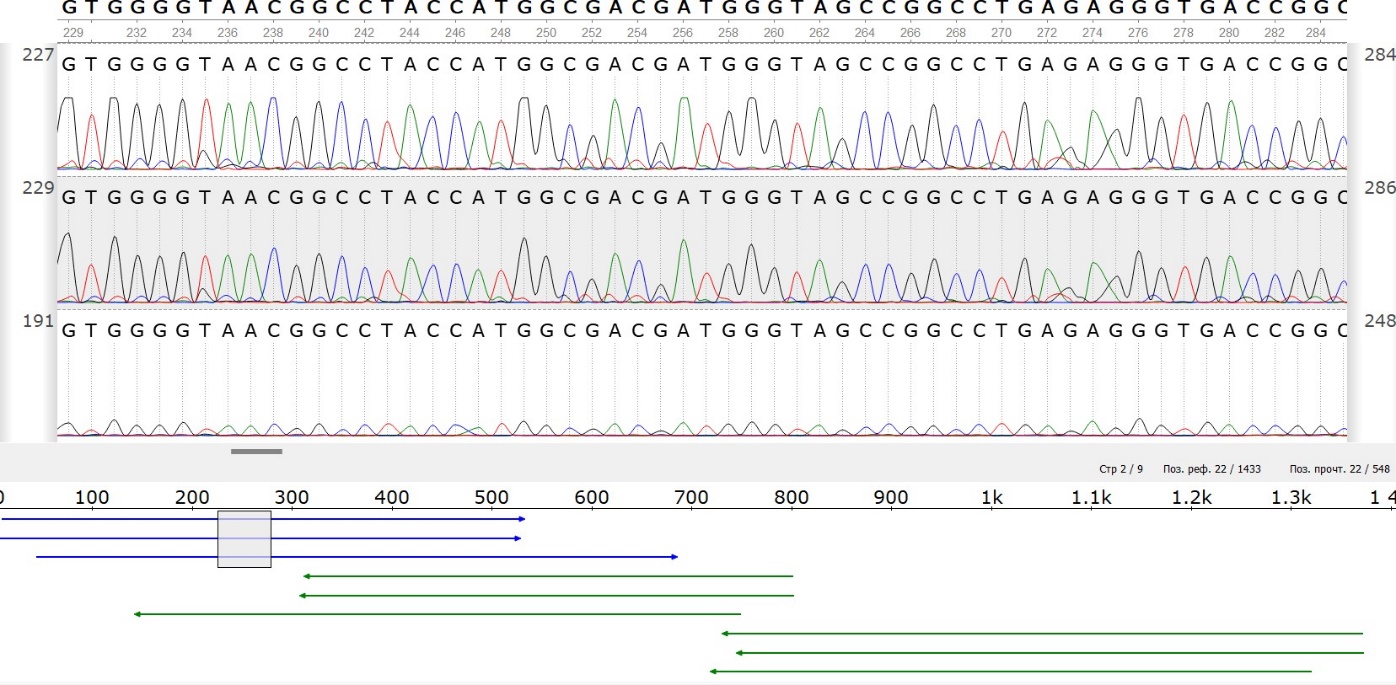

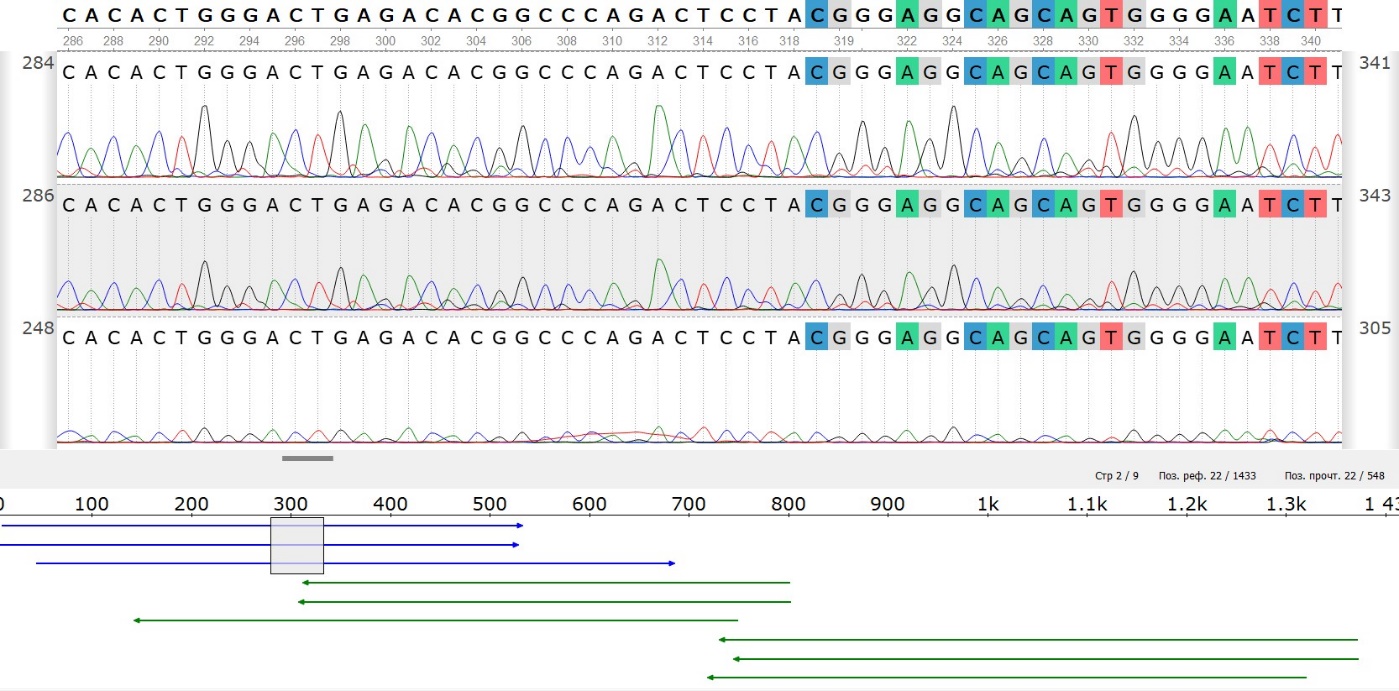


1 27F

2 27F

3 27F

consensus

1 27F

2 27F

3 27F


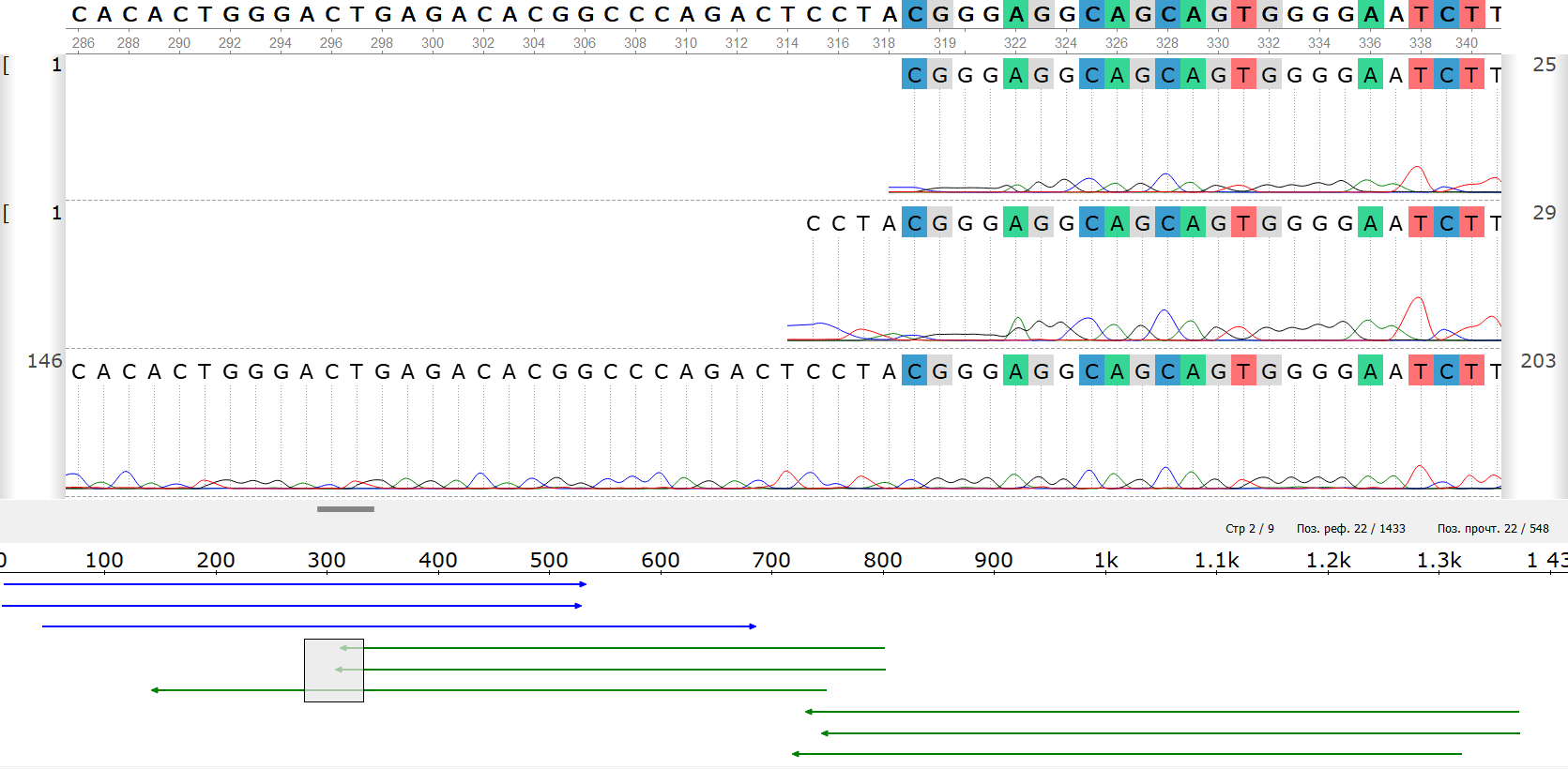
consensus

1 907R

2 907R

3 907R


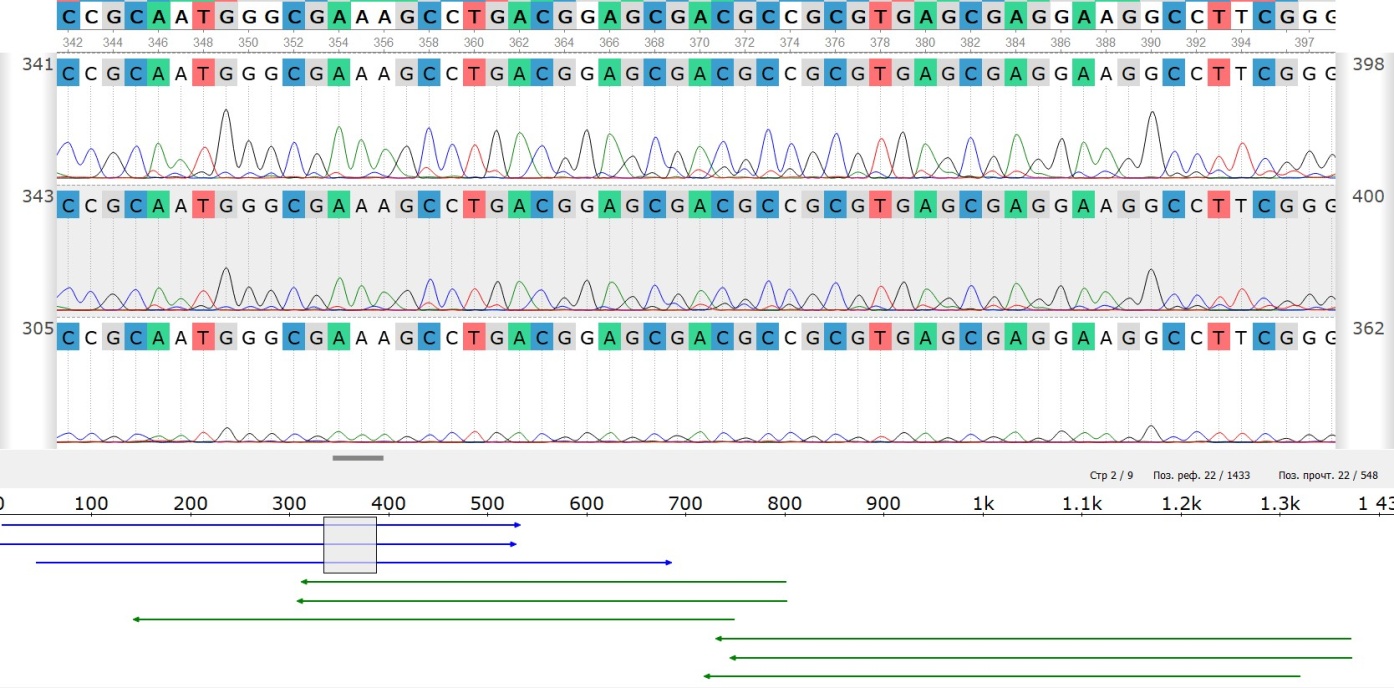
consensus

1 27F

2 27F

3 27F


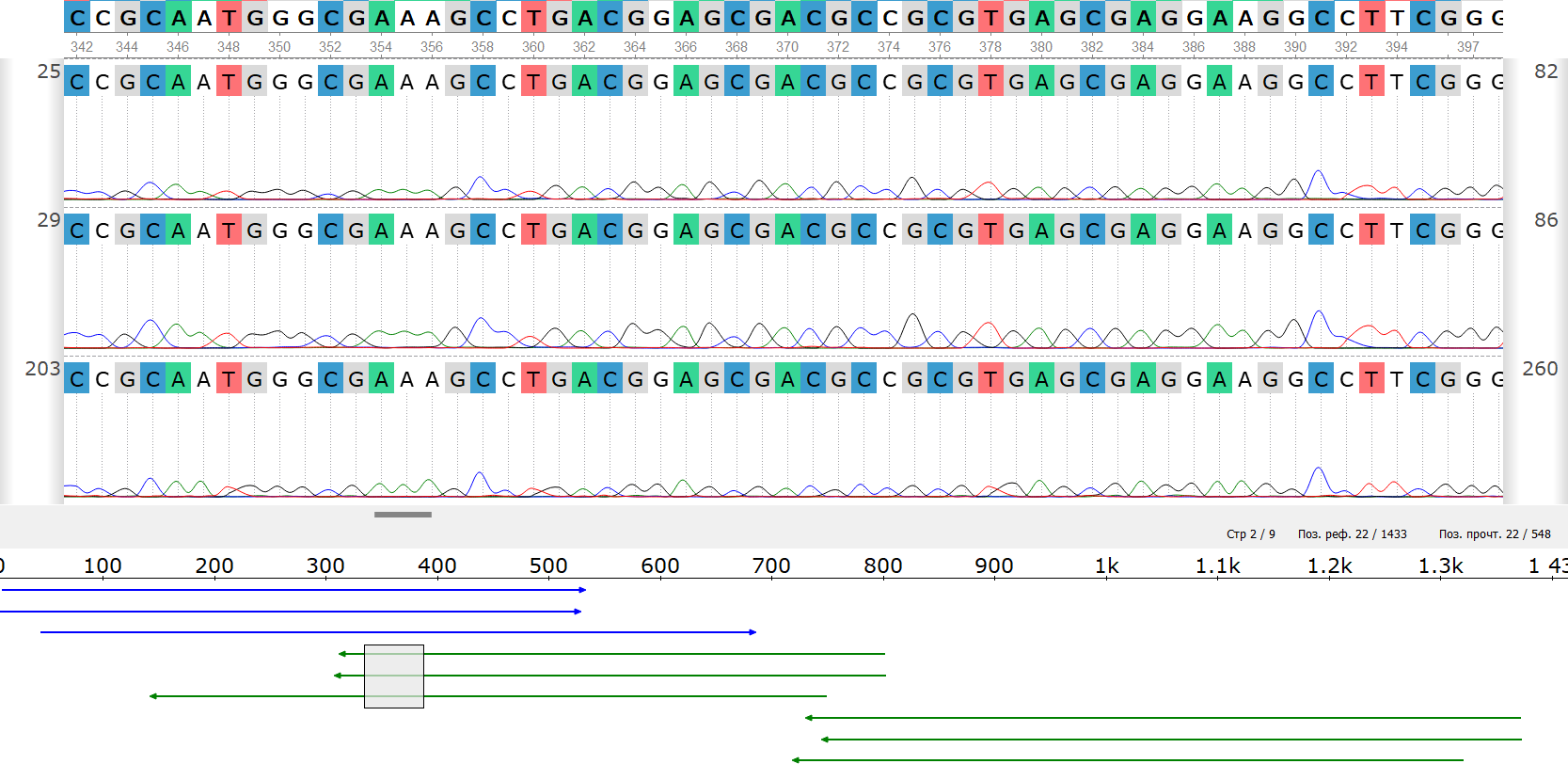
consensus

1 907R

2 907R

3 907R


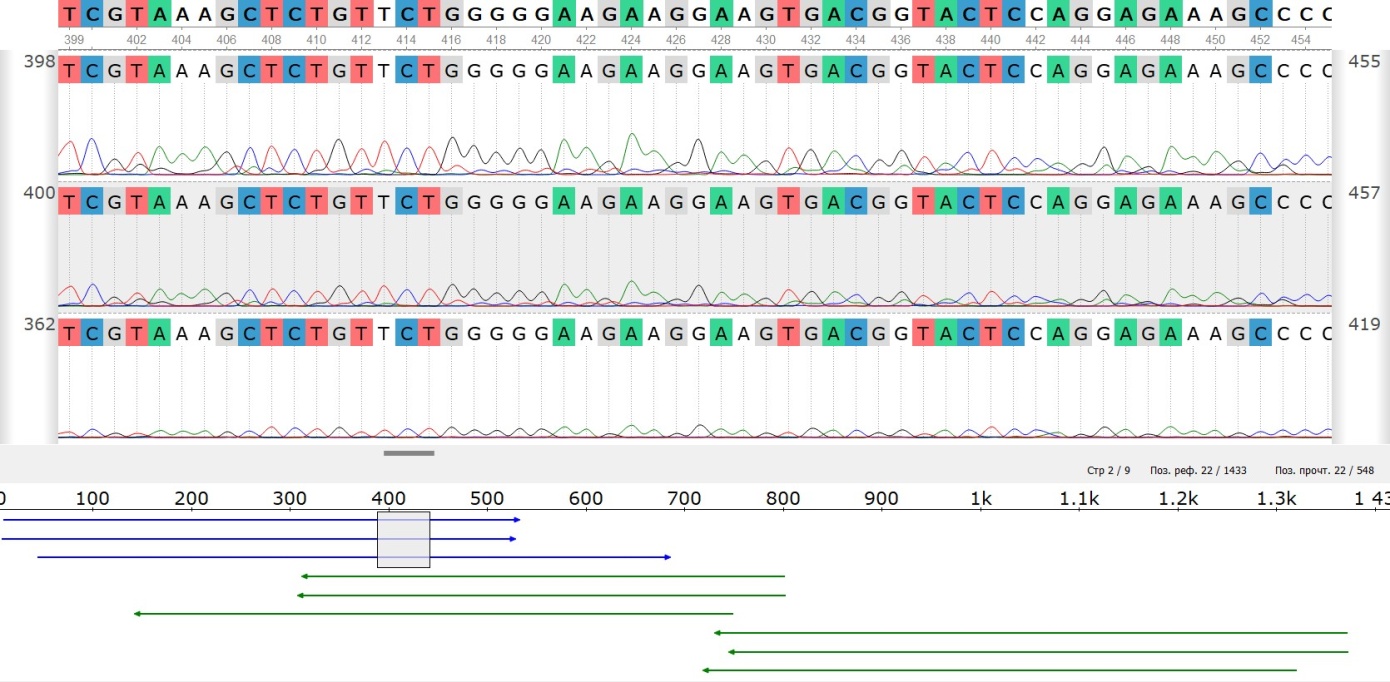
consensus

1 27F

2 27F

3 27F

consensus


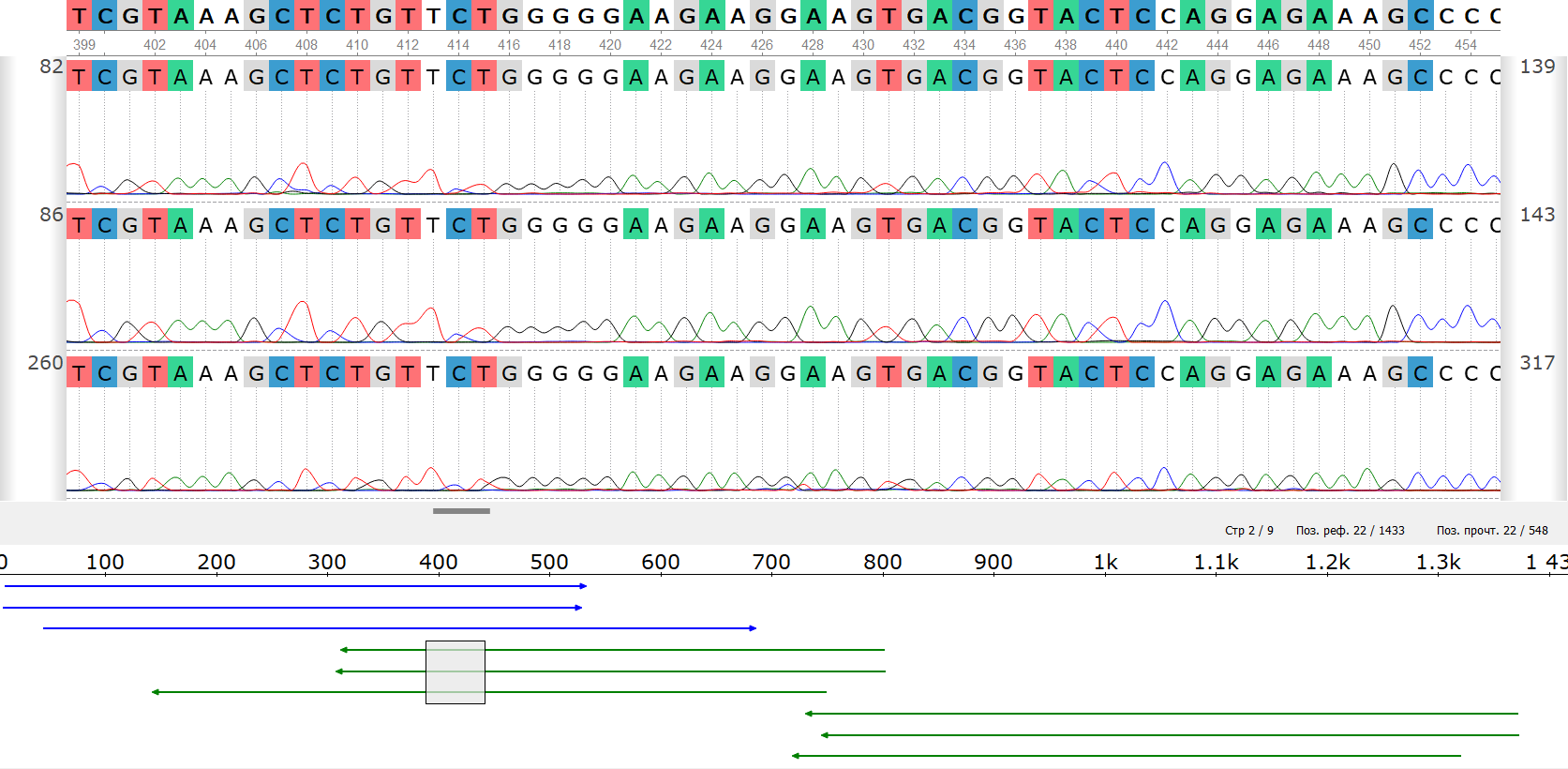

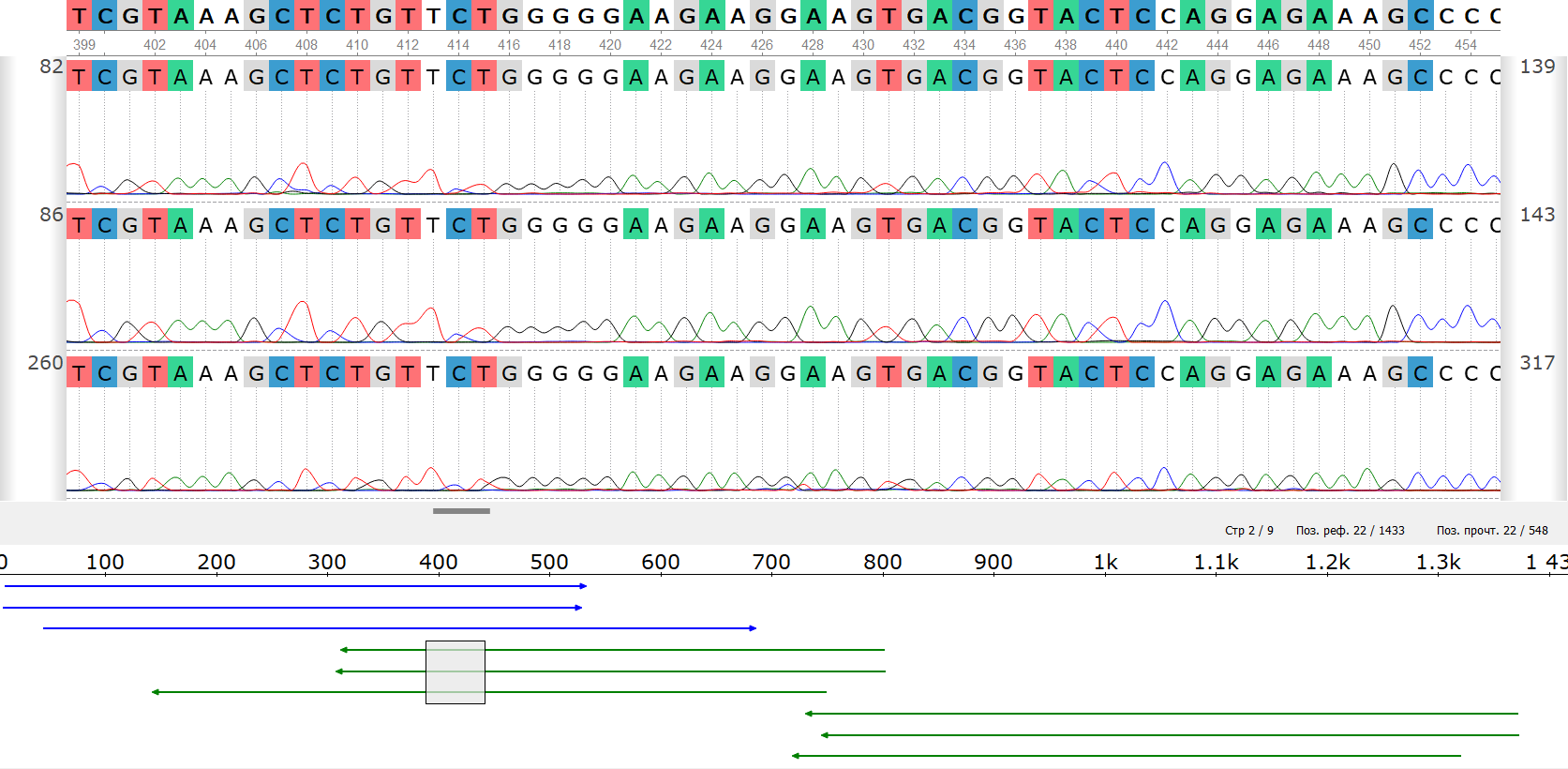


1 907R

2 907R

3 907R


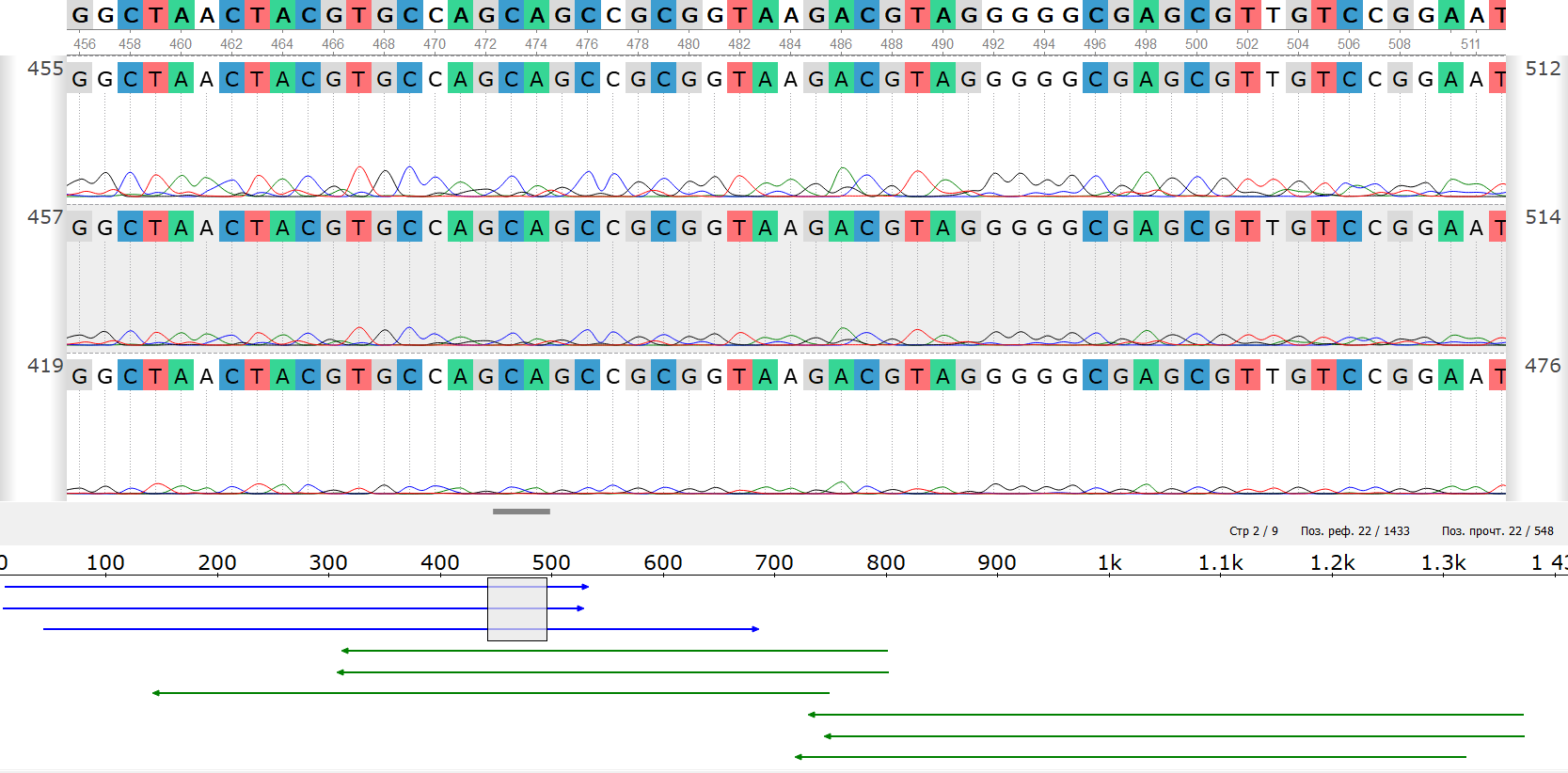
consensus

1 27F

2 27F

3 27F

consensus


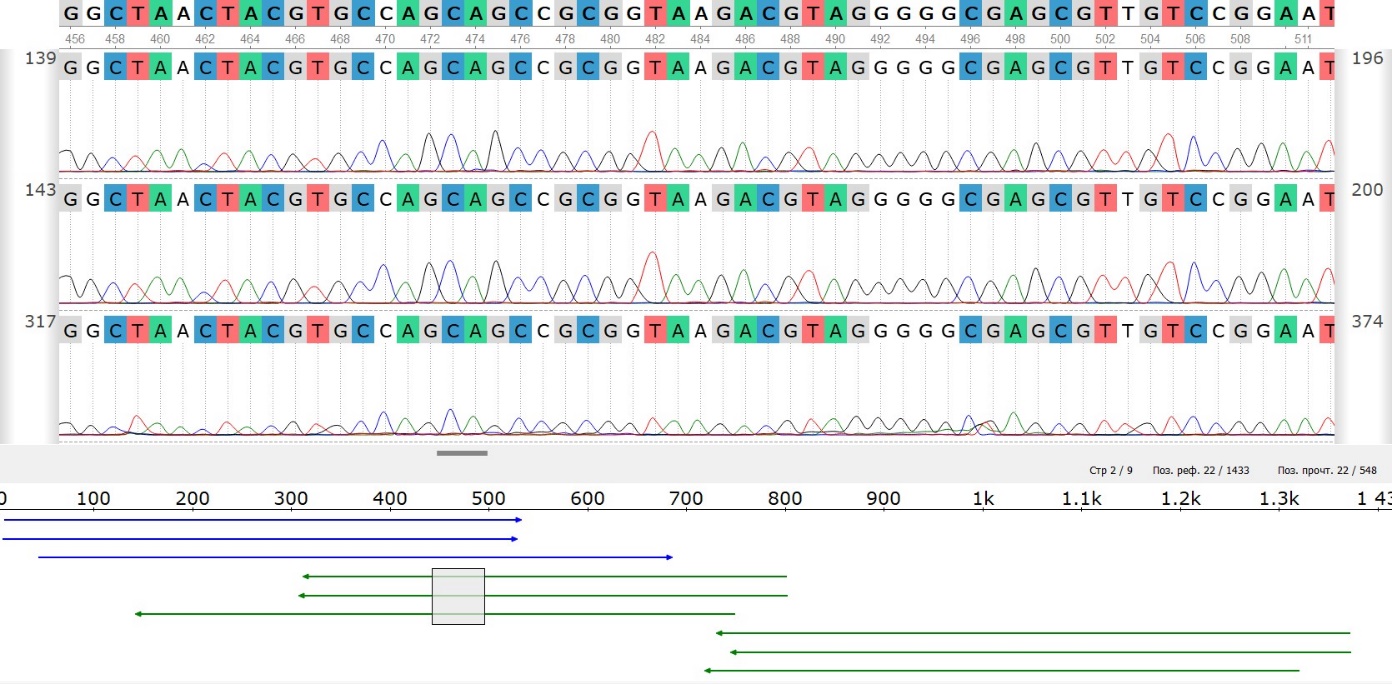

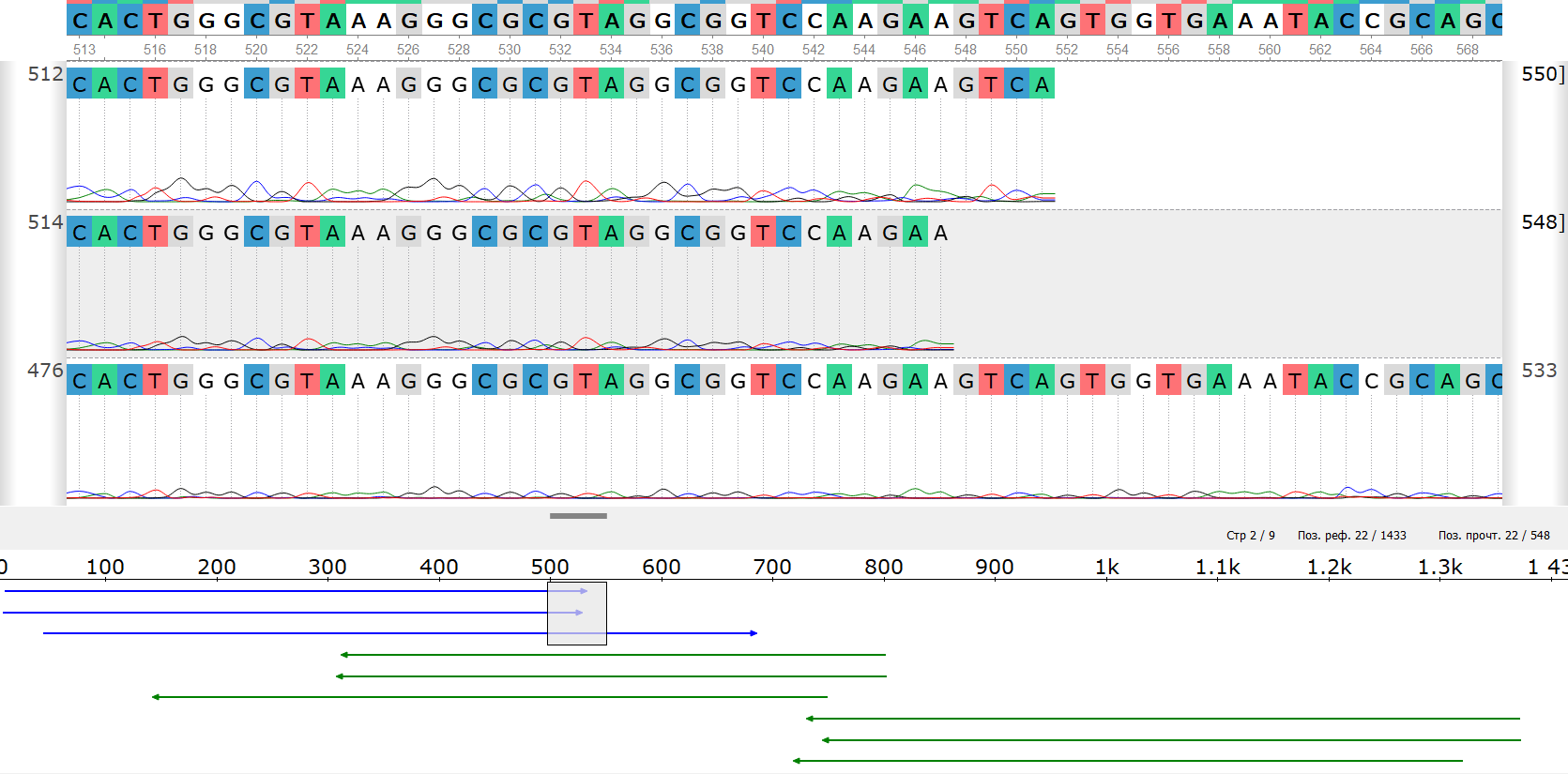

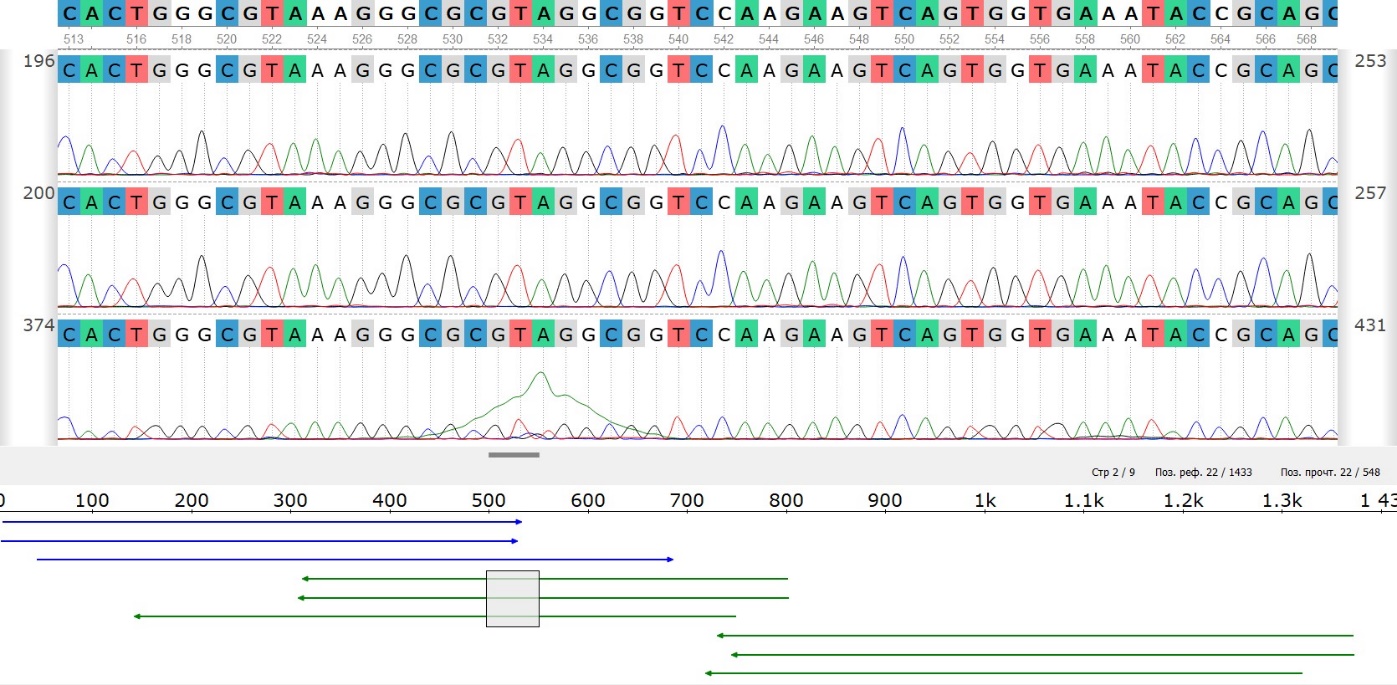


1 907R

2 907R

3 907R

consensus

1 27F

2 27F

3 27F

consensus

1 907R

2 907R

3 907R


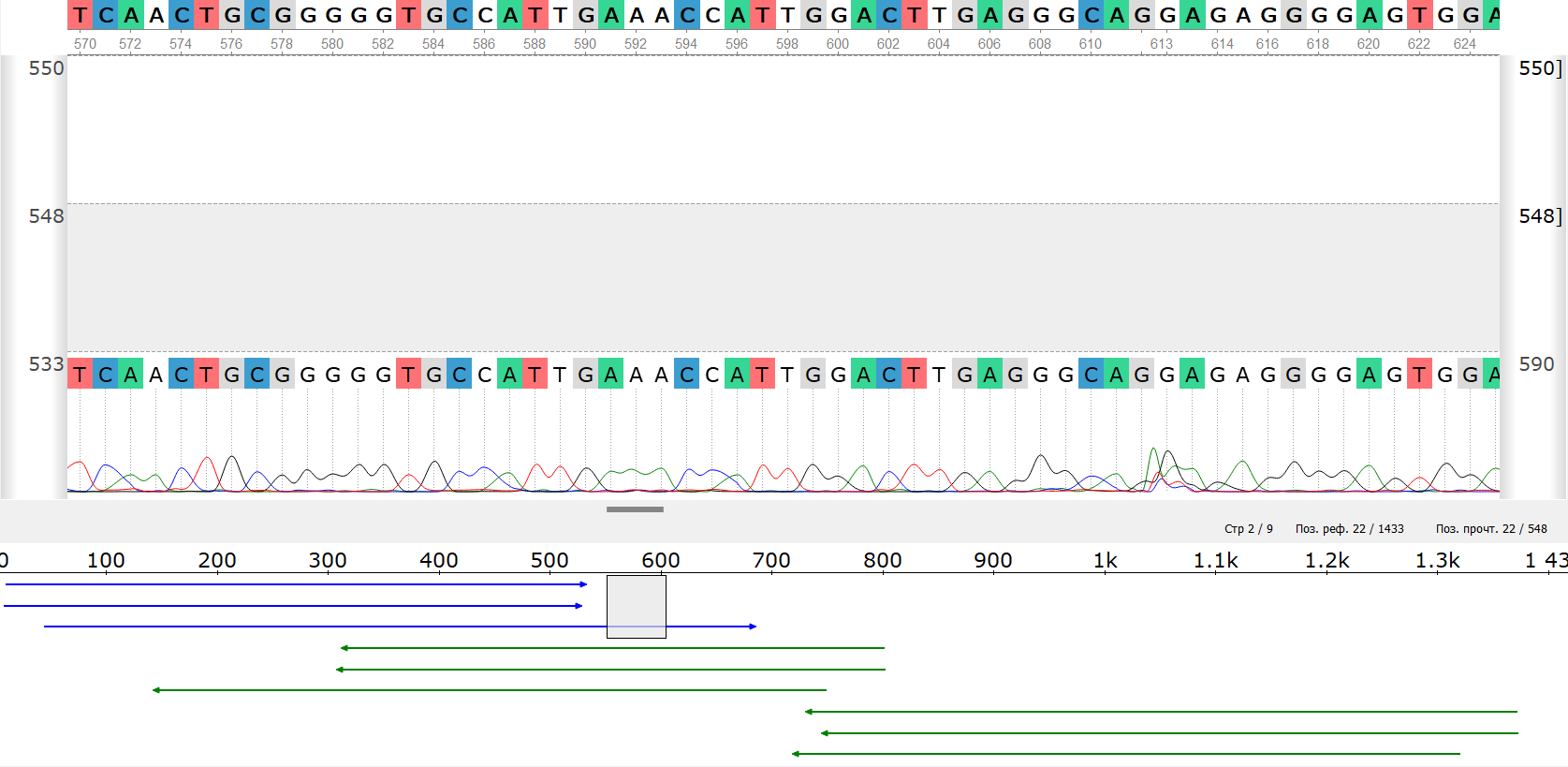
consensus

1 27F

2 27F

3 27F


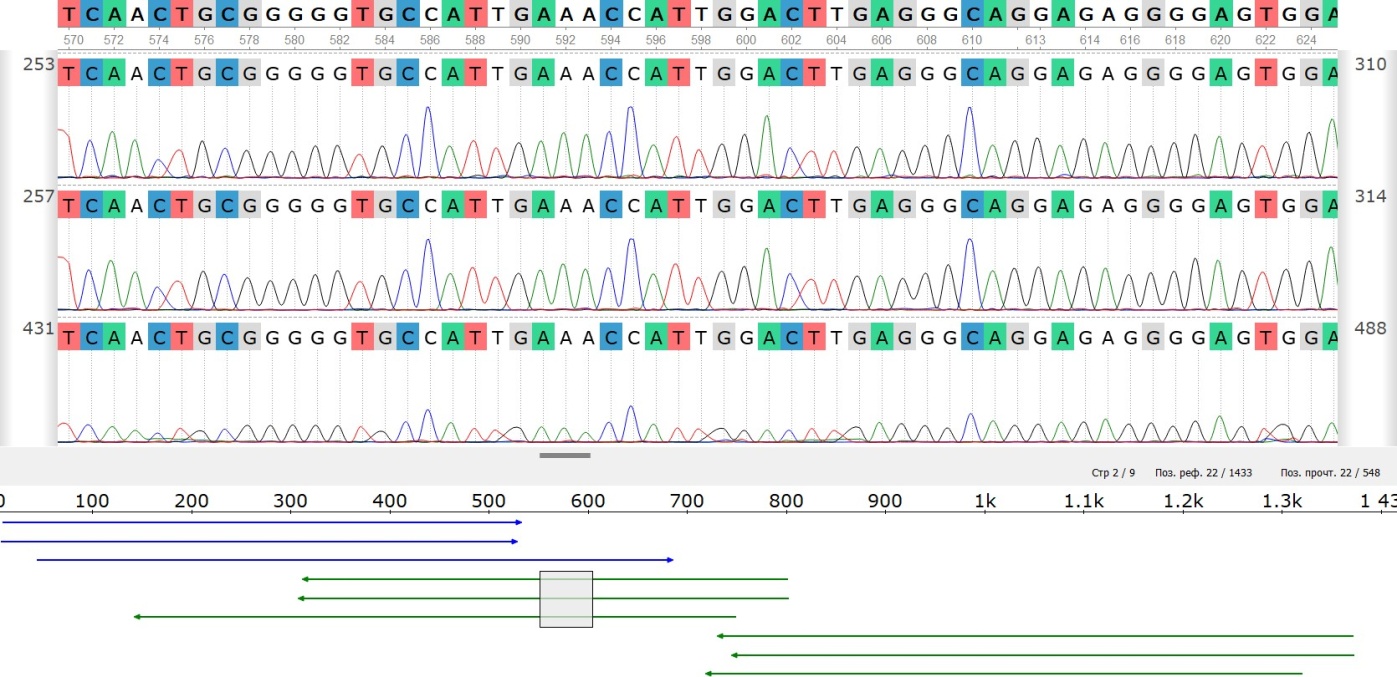
consensus

1 907R

2 907R

3 907R


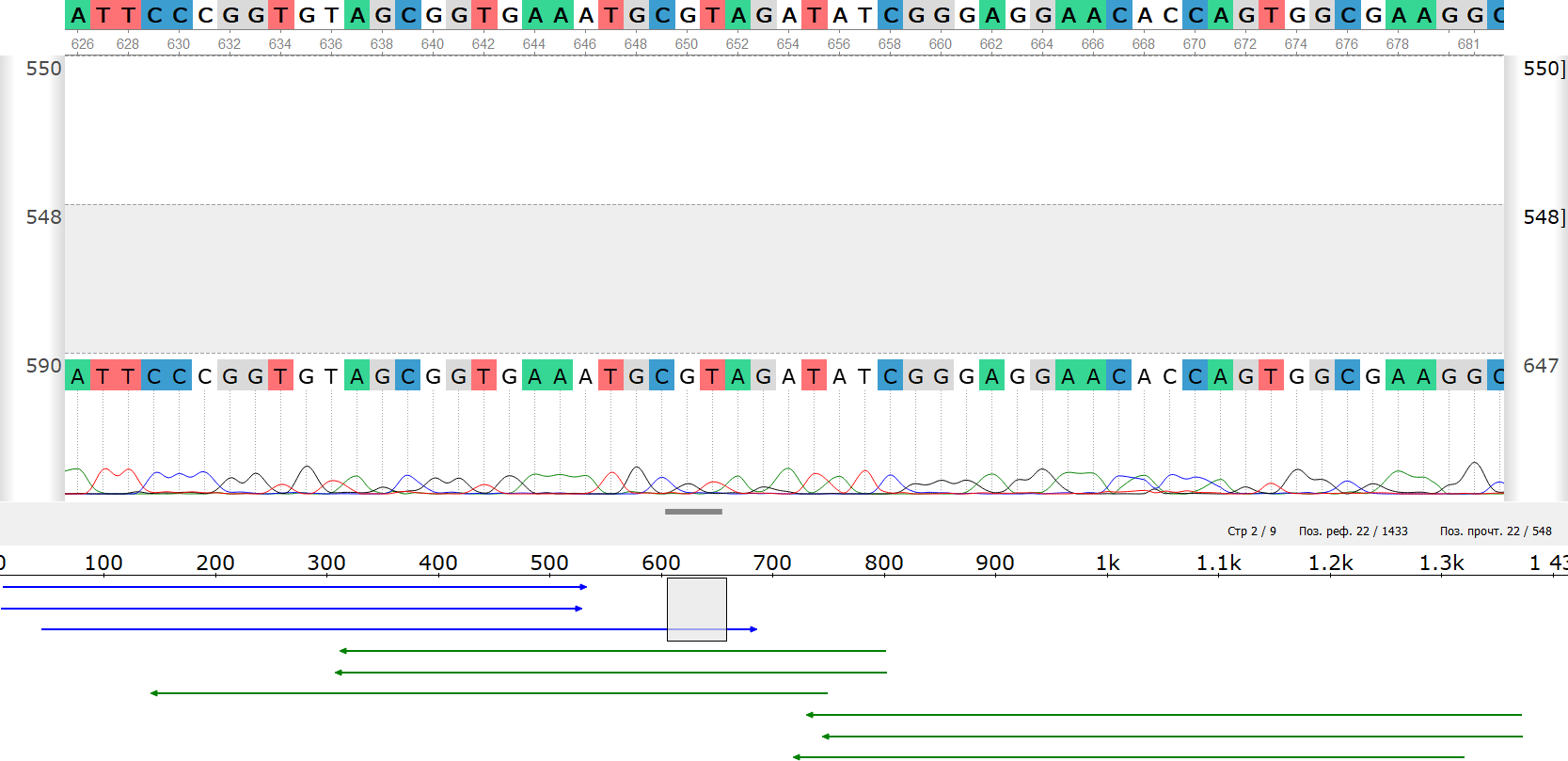
consensus

1 27F

2 27F

3 27F


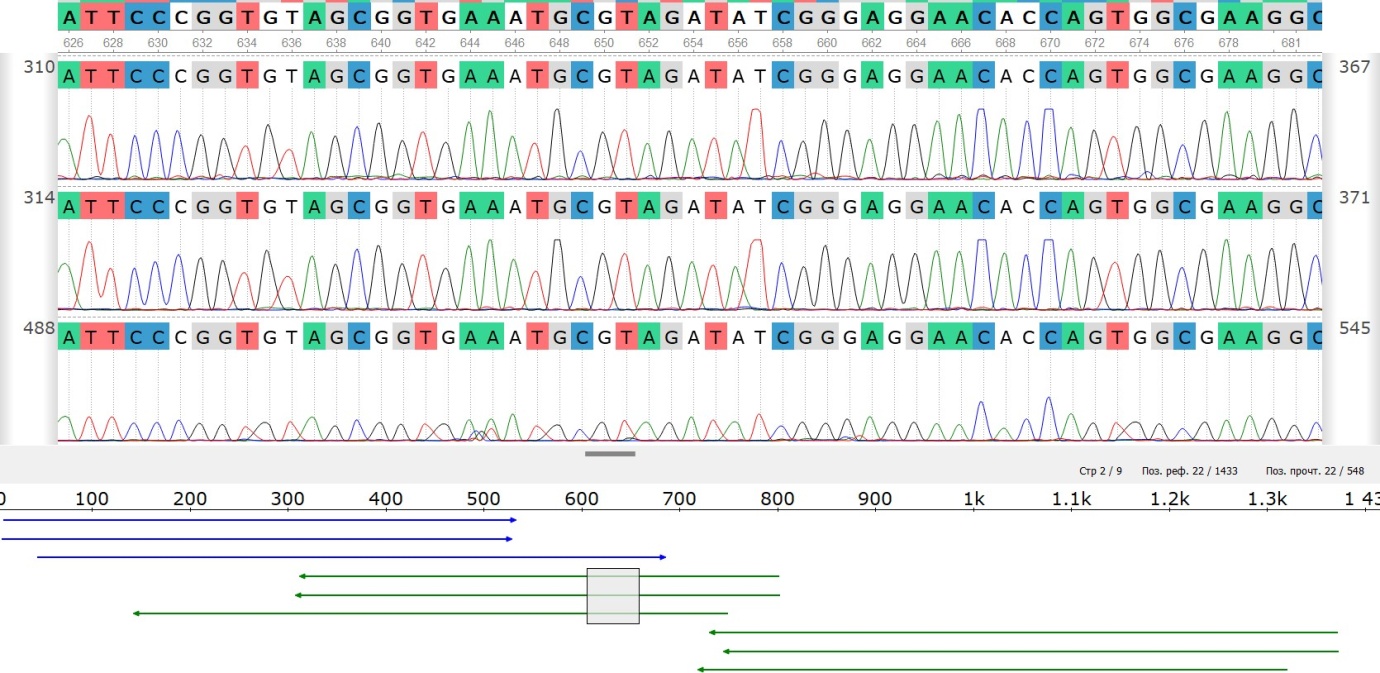
consensus

1 907R

2 907R

3 907R


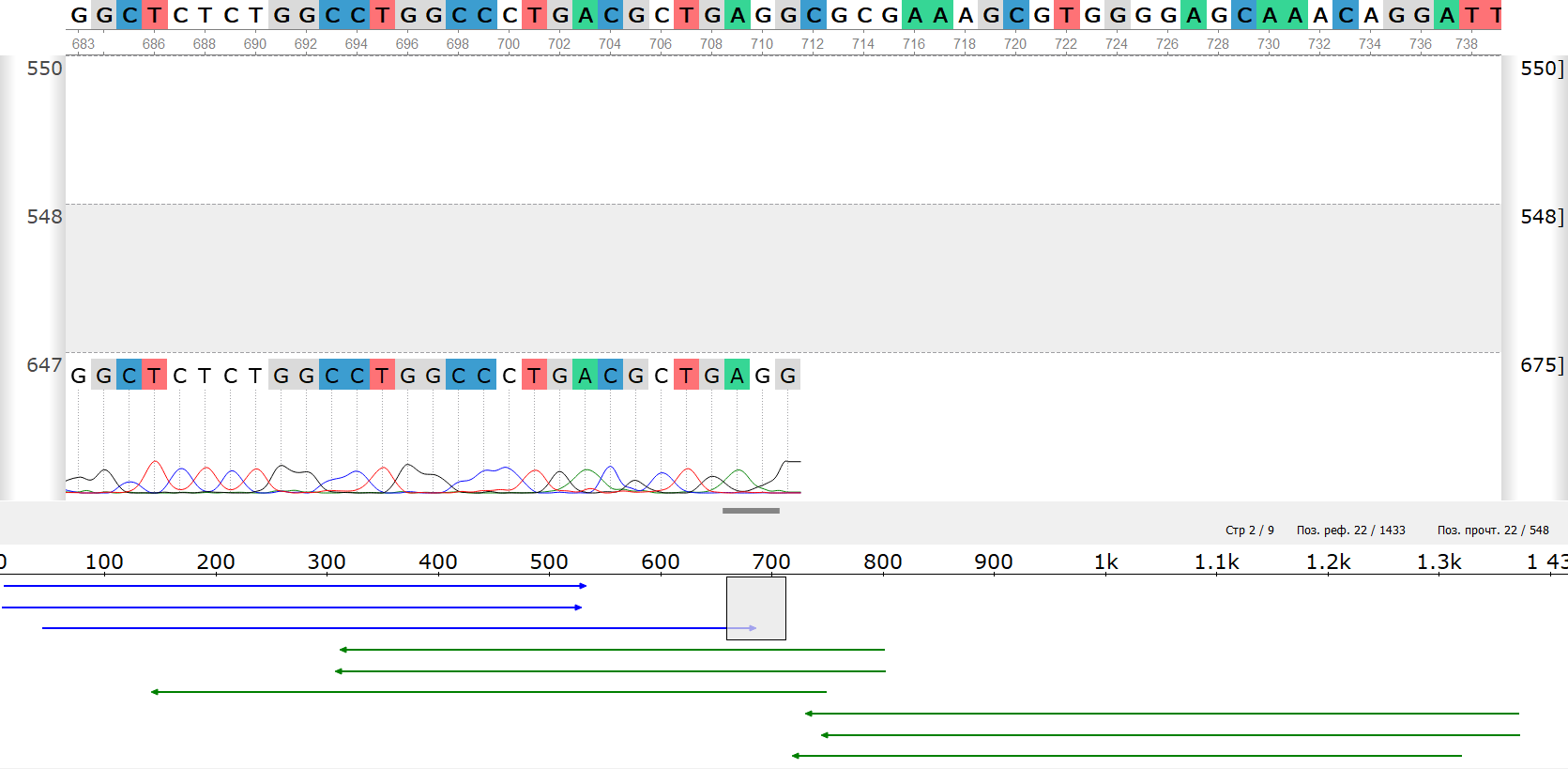
consensus

1 27F

2 27F

3 27F


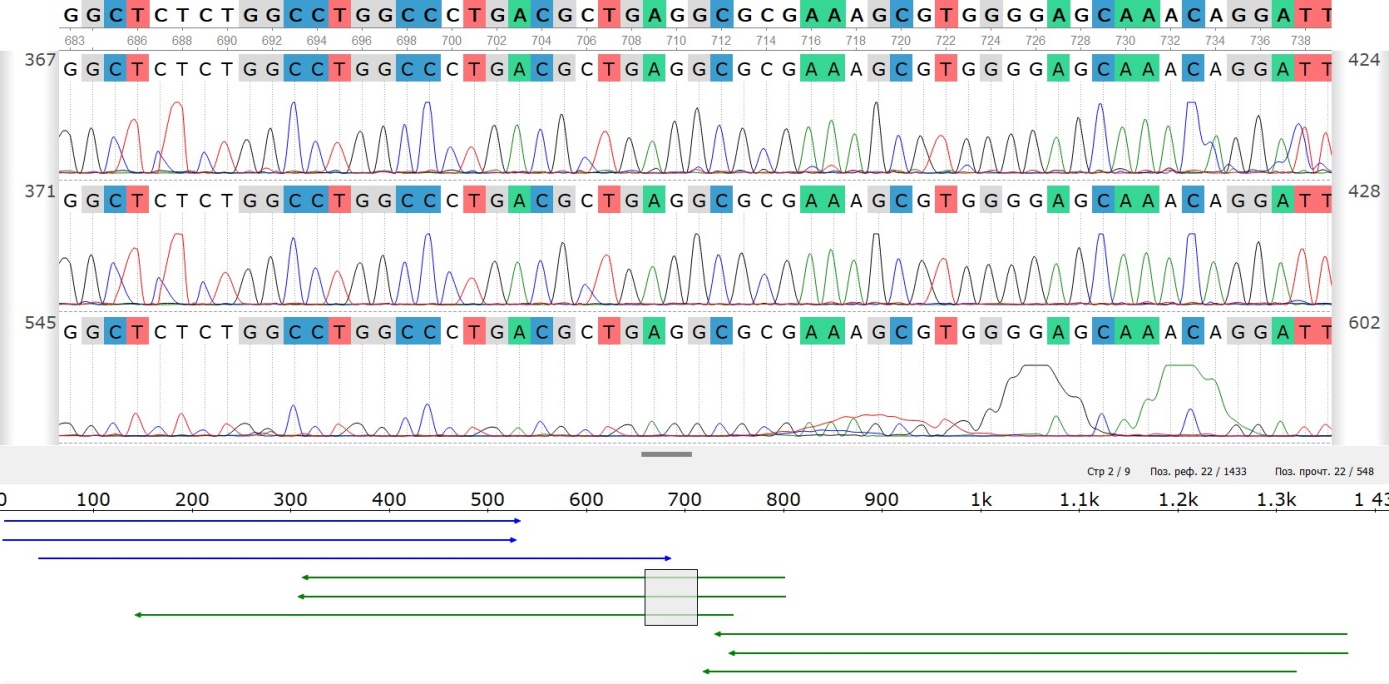
consensus

1 907R

2 907R

3 907R


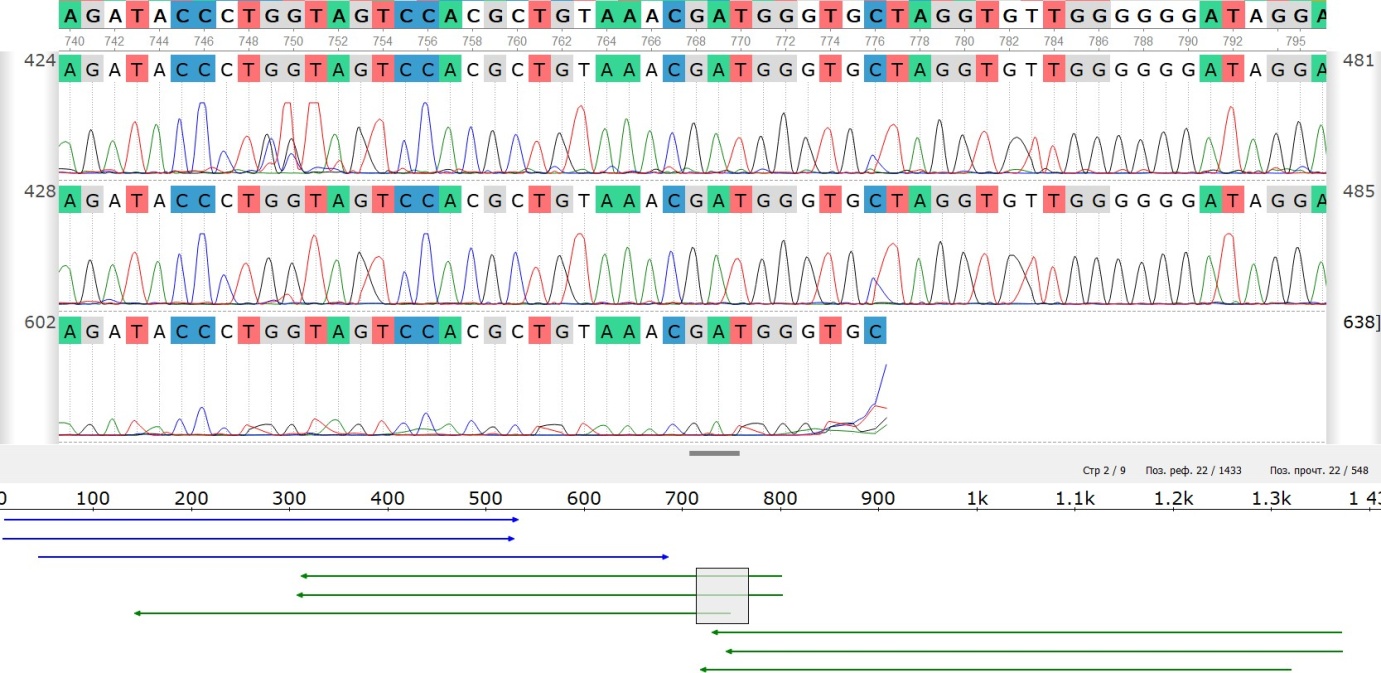
consensus

1 907R

2 907R

3 907R


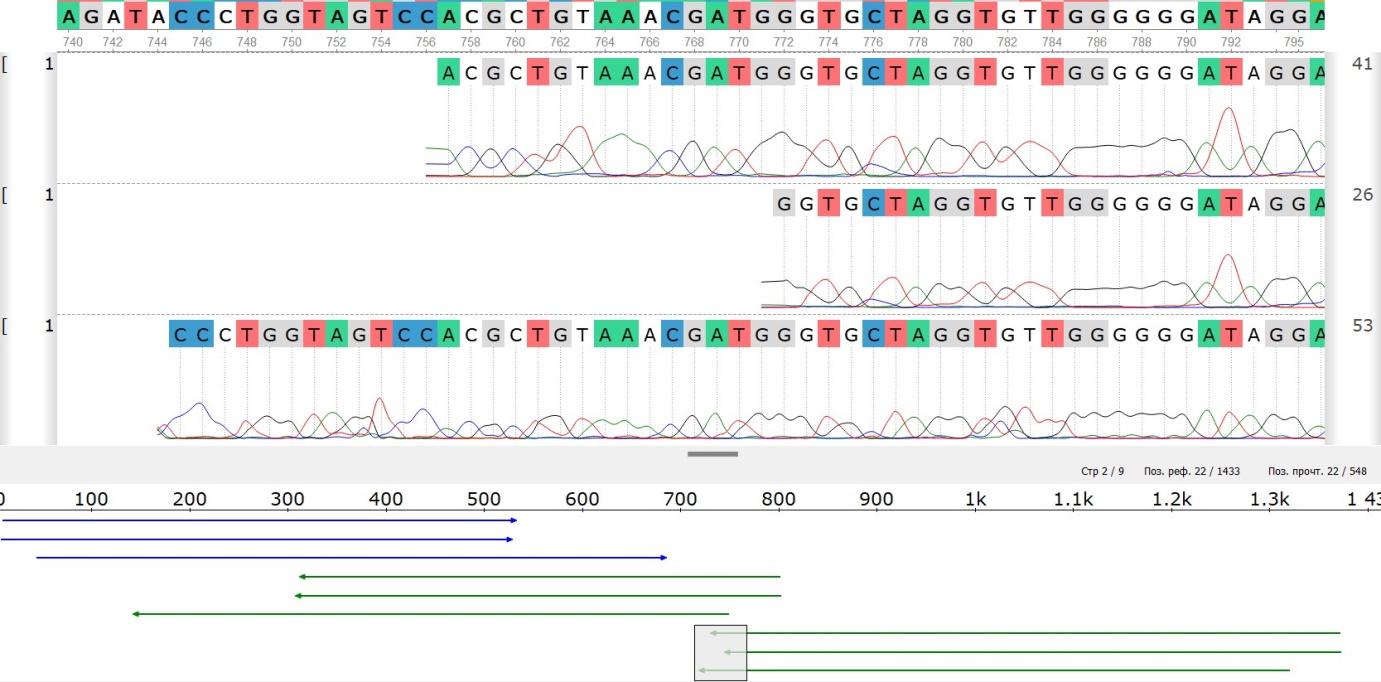
consensus

1 1492R

2 1492R

3 1492R


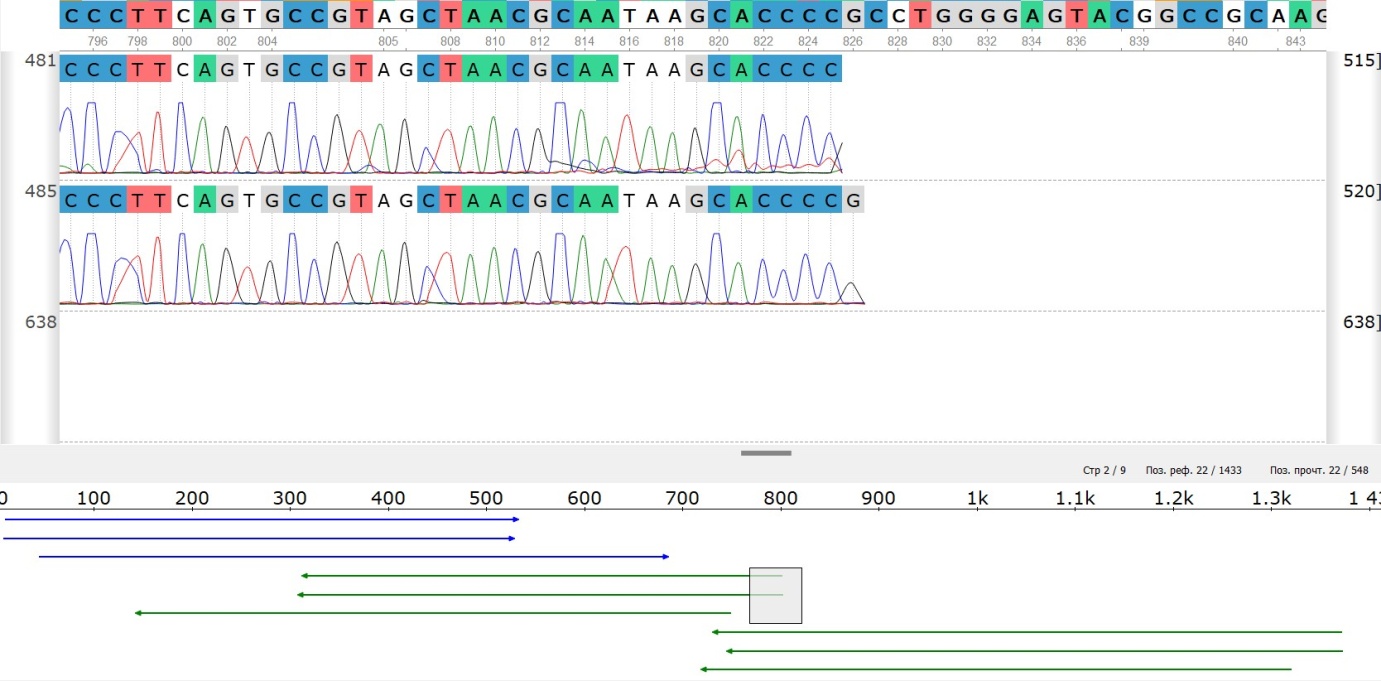
consensus

1 907R

2 907R

3 907R


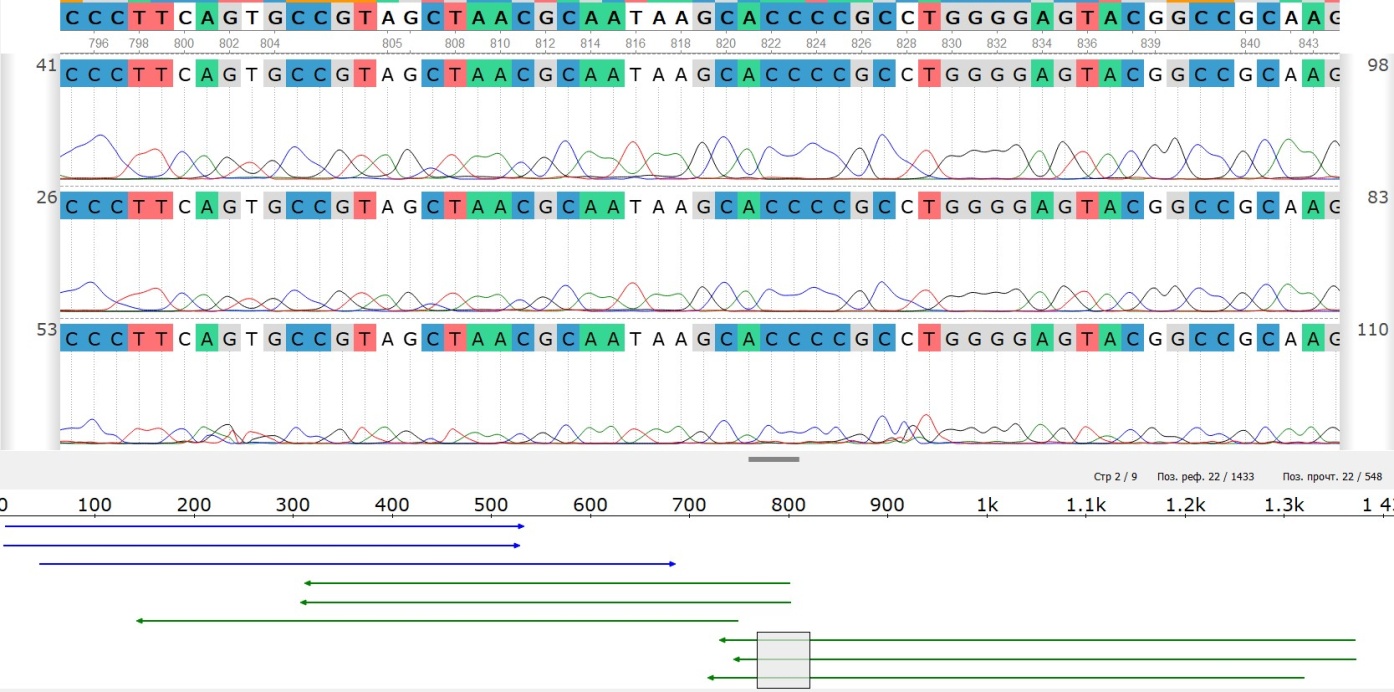
consensus

1 1492R

2 1492R

3 1492R


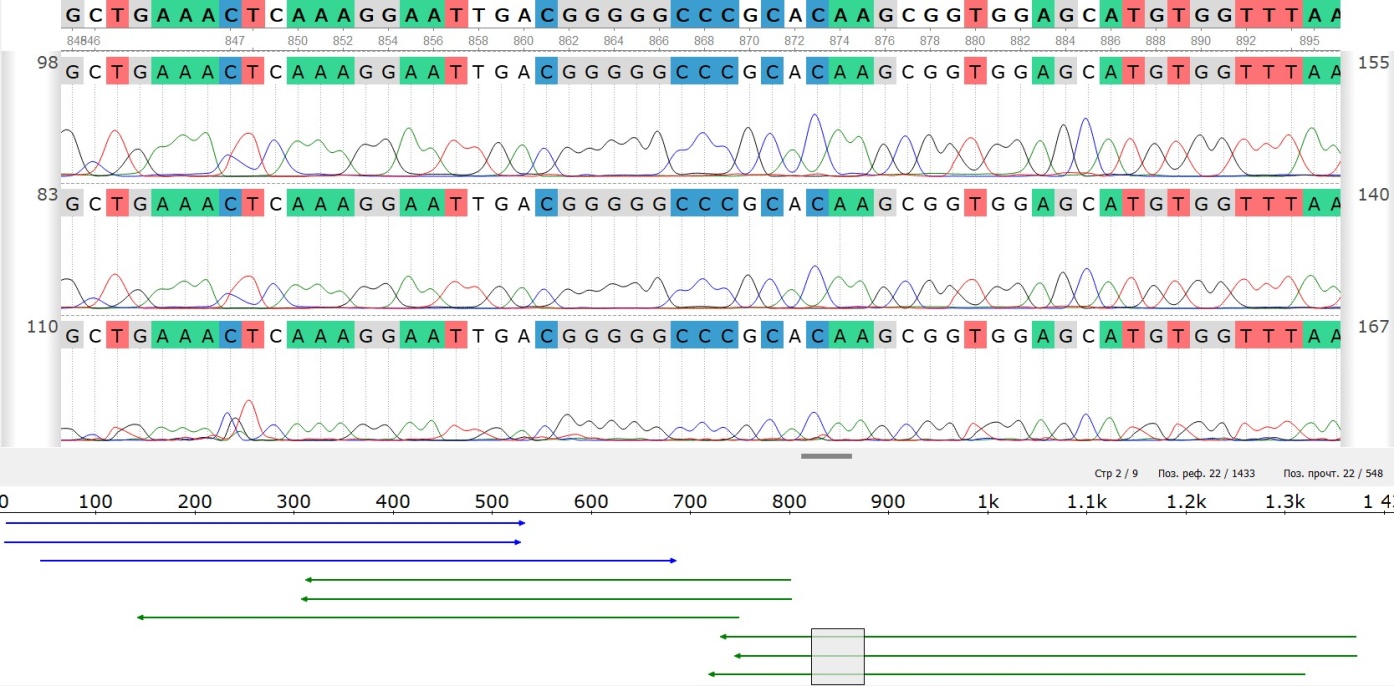
consensus

1 1492R

2 1492R

3 1492R


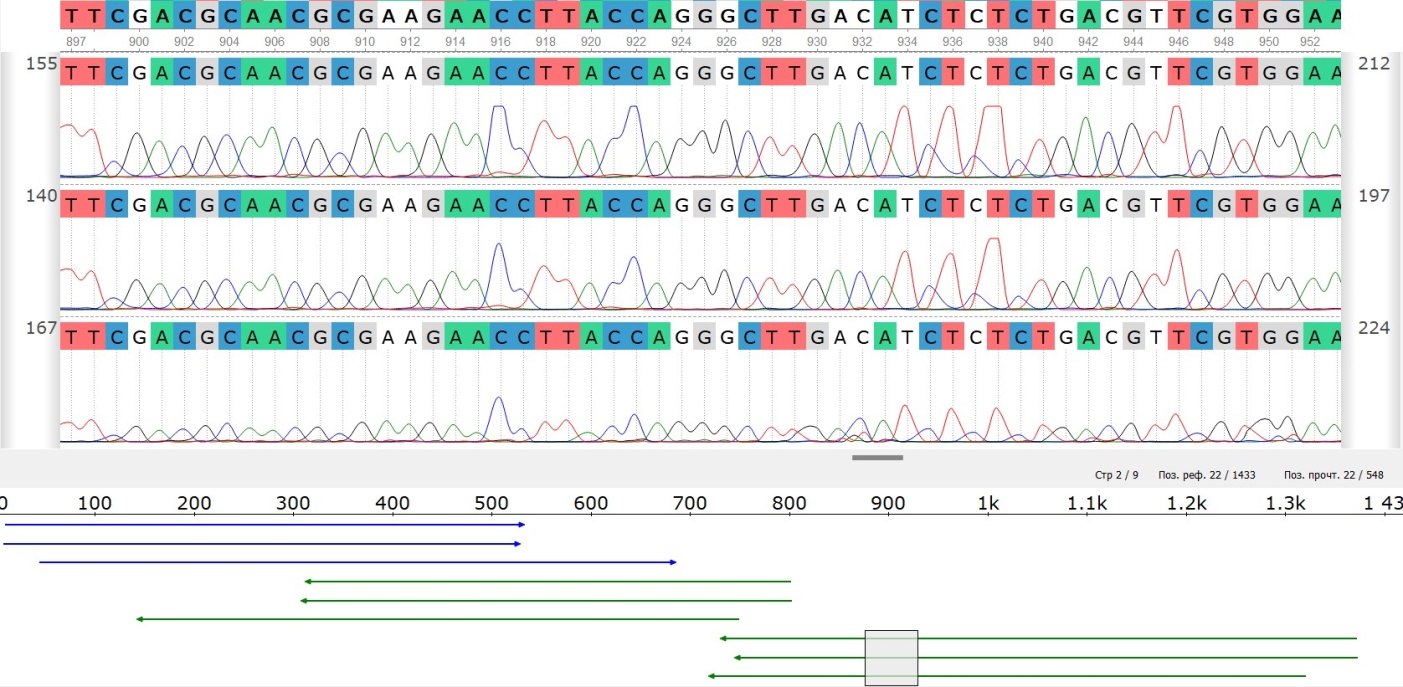
consensus

1 1492R

2 1492R

3 1492R


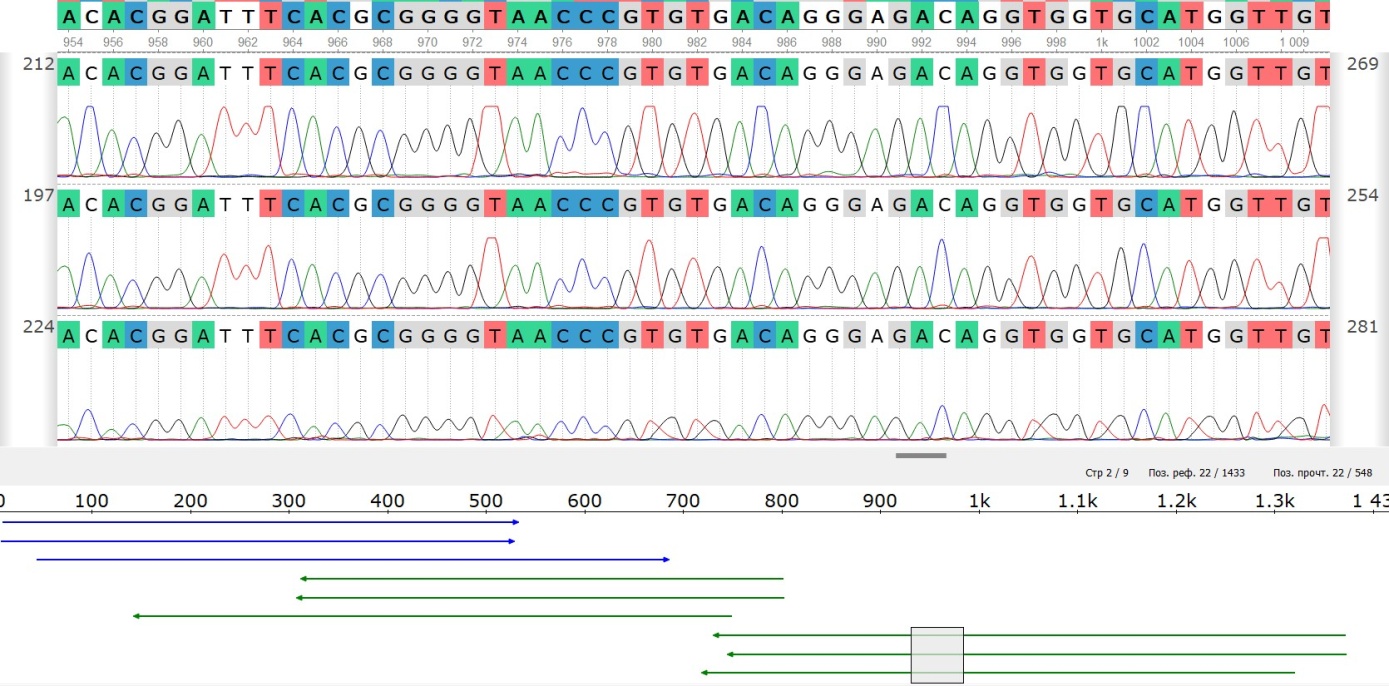
consensus

1 1492R

2 1492R

3 1492R


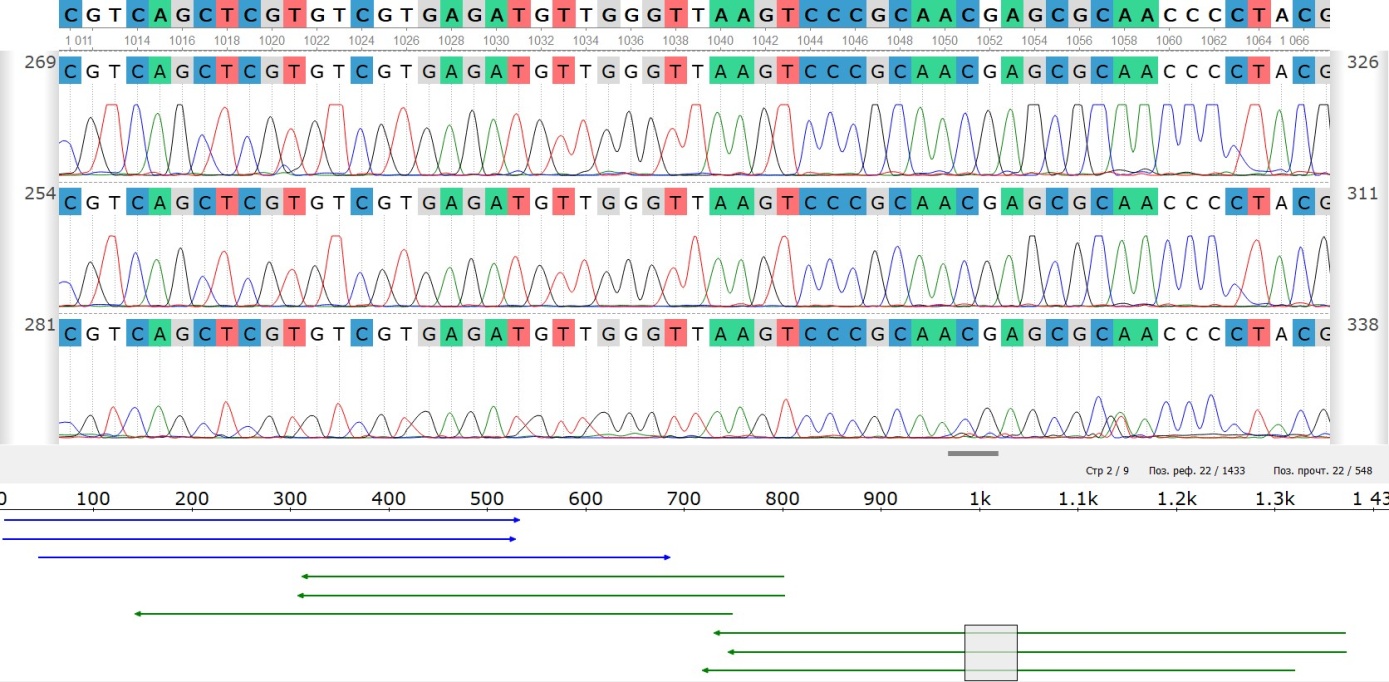
consensus

1 1492R

2 1492R

3 1492R


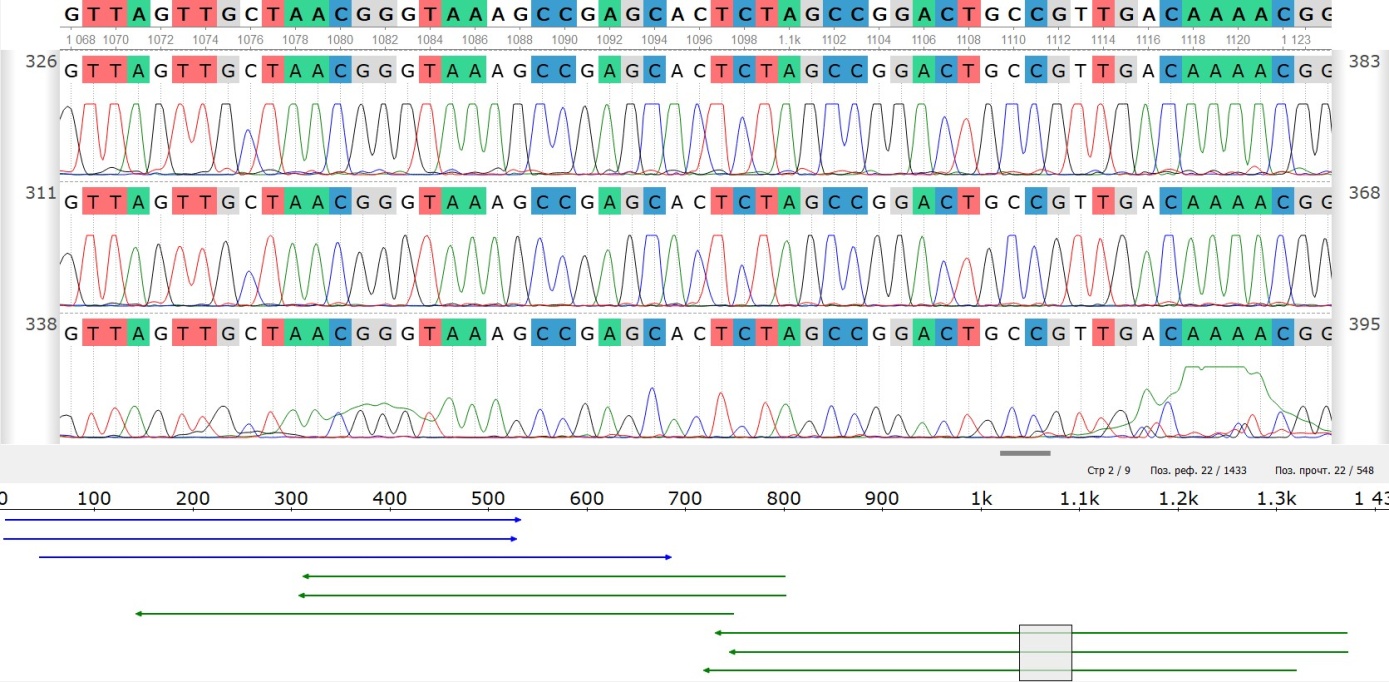
consensus

1 1492R

2 1492R

3 1492R


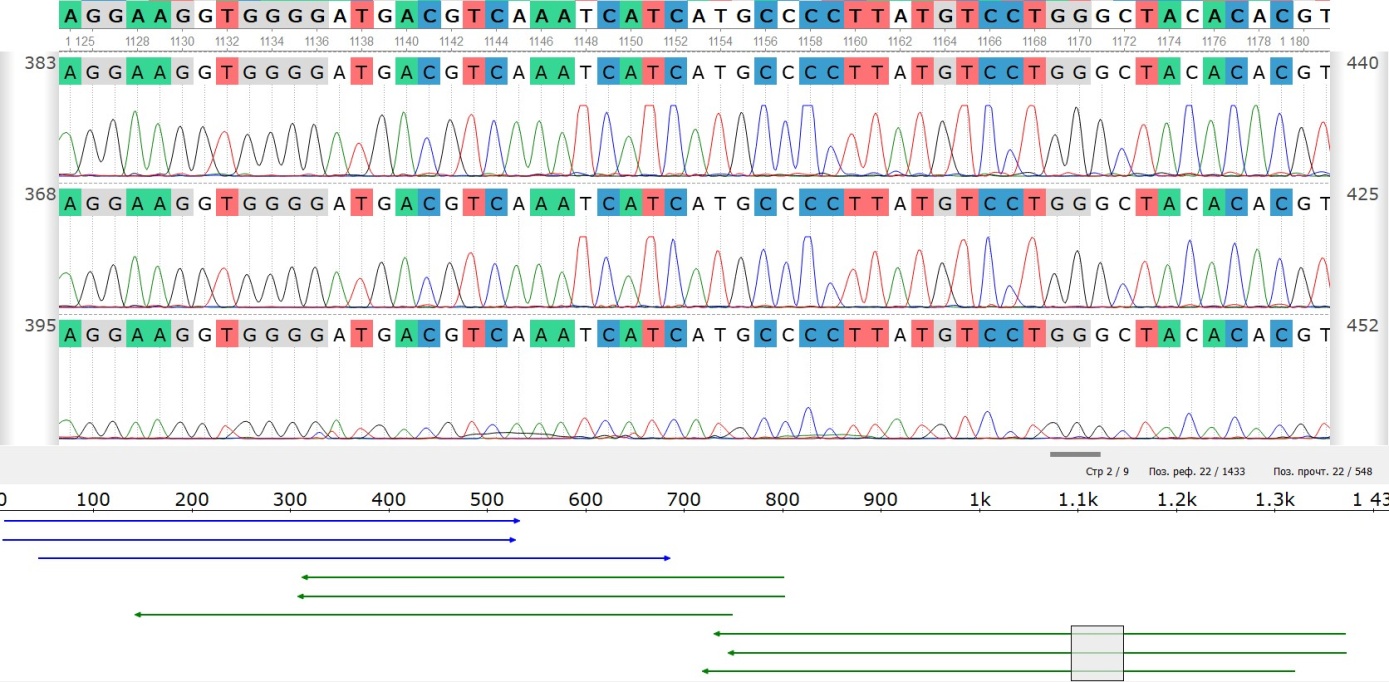
consensus

1 1492R

2 1492R

3 1492R


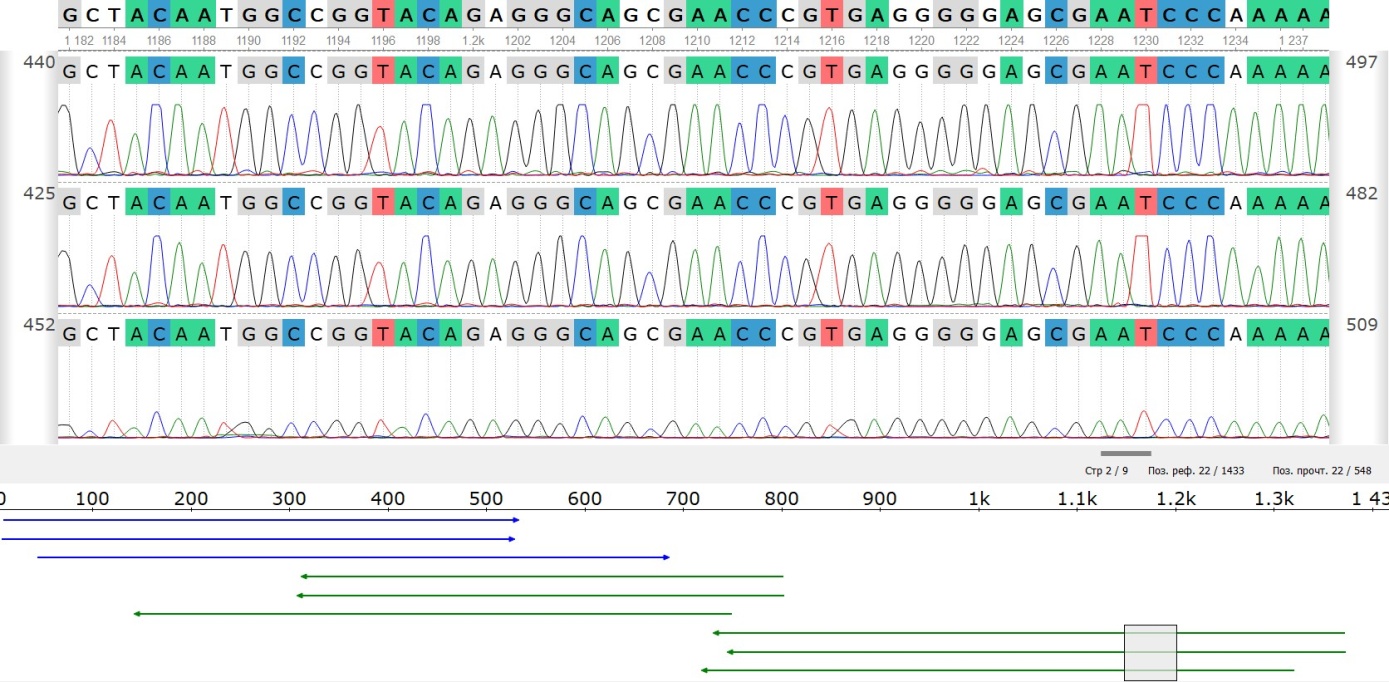
consensus

1 1492R

2 1492R

3 1492R


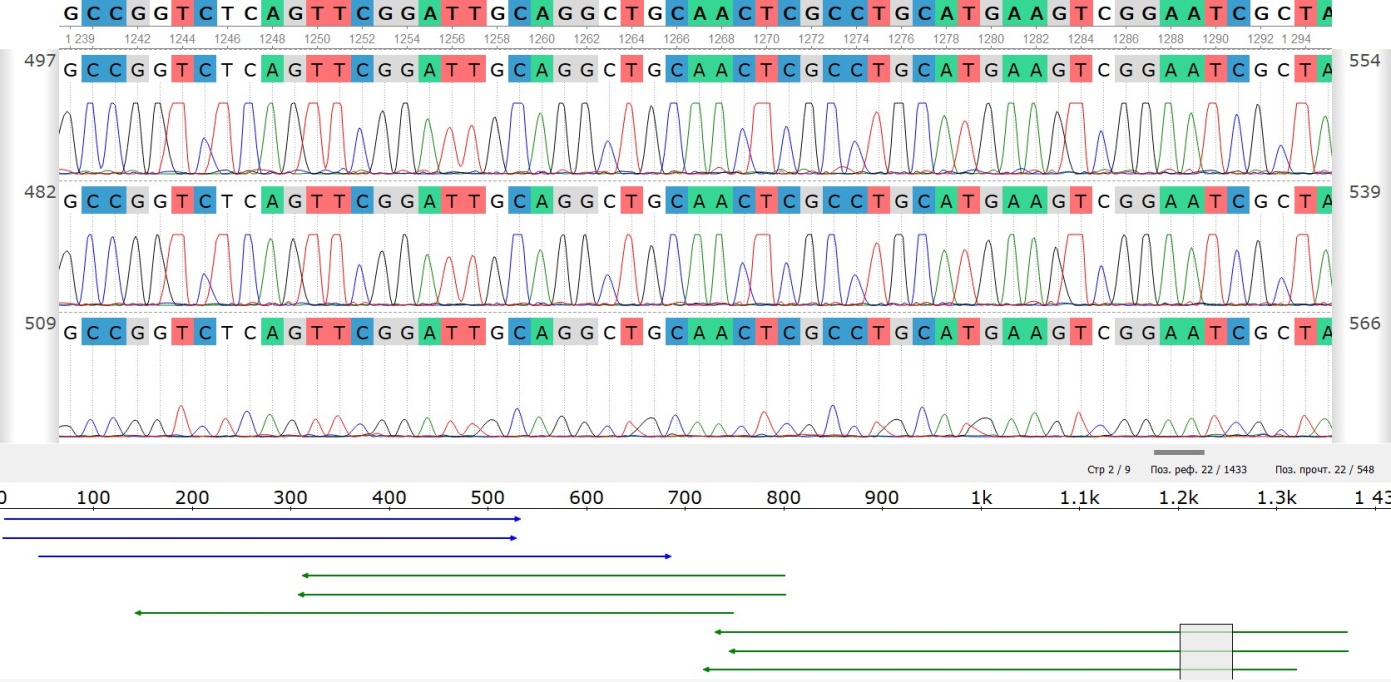
consensus

1 1492R

2 1492R

3 1492R


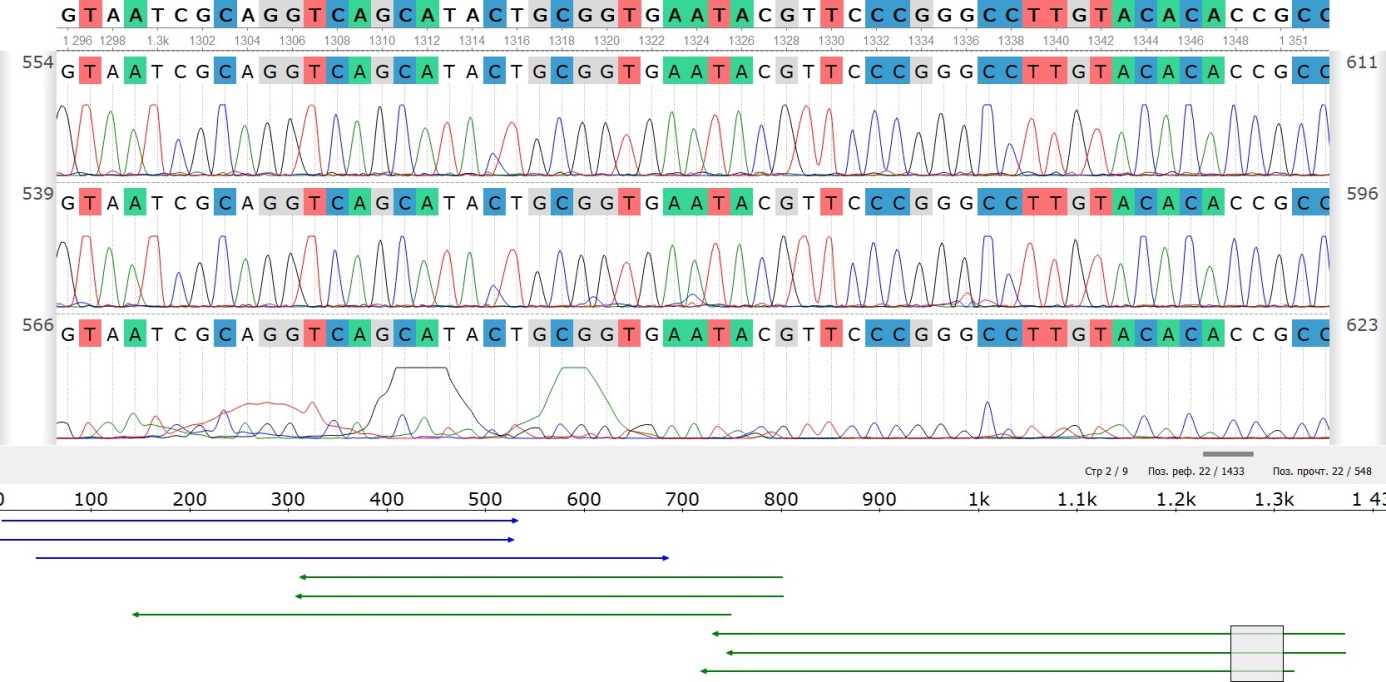
consensus

1 1492R

2 1492R

3 1492R


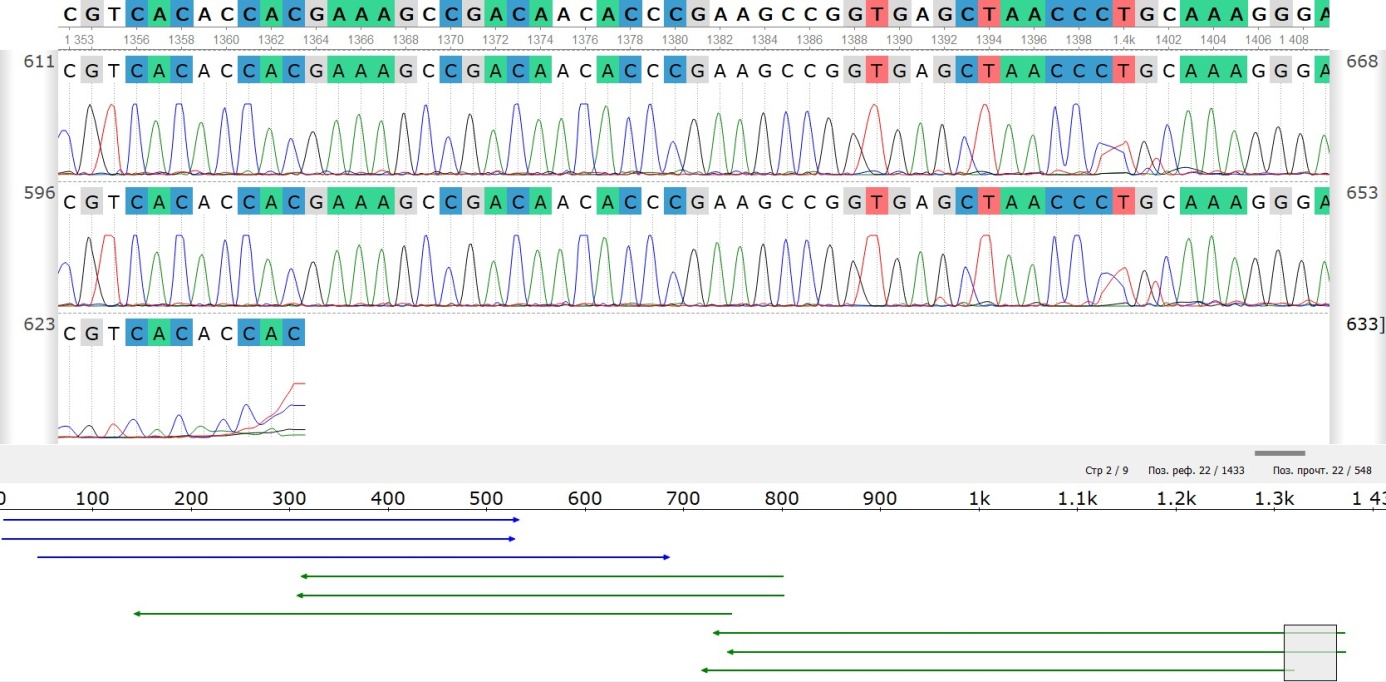
consensus

1 1492R

2 1492R

3 1492R


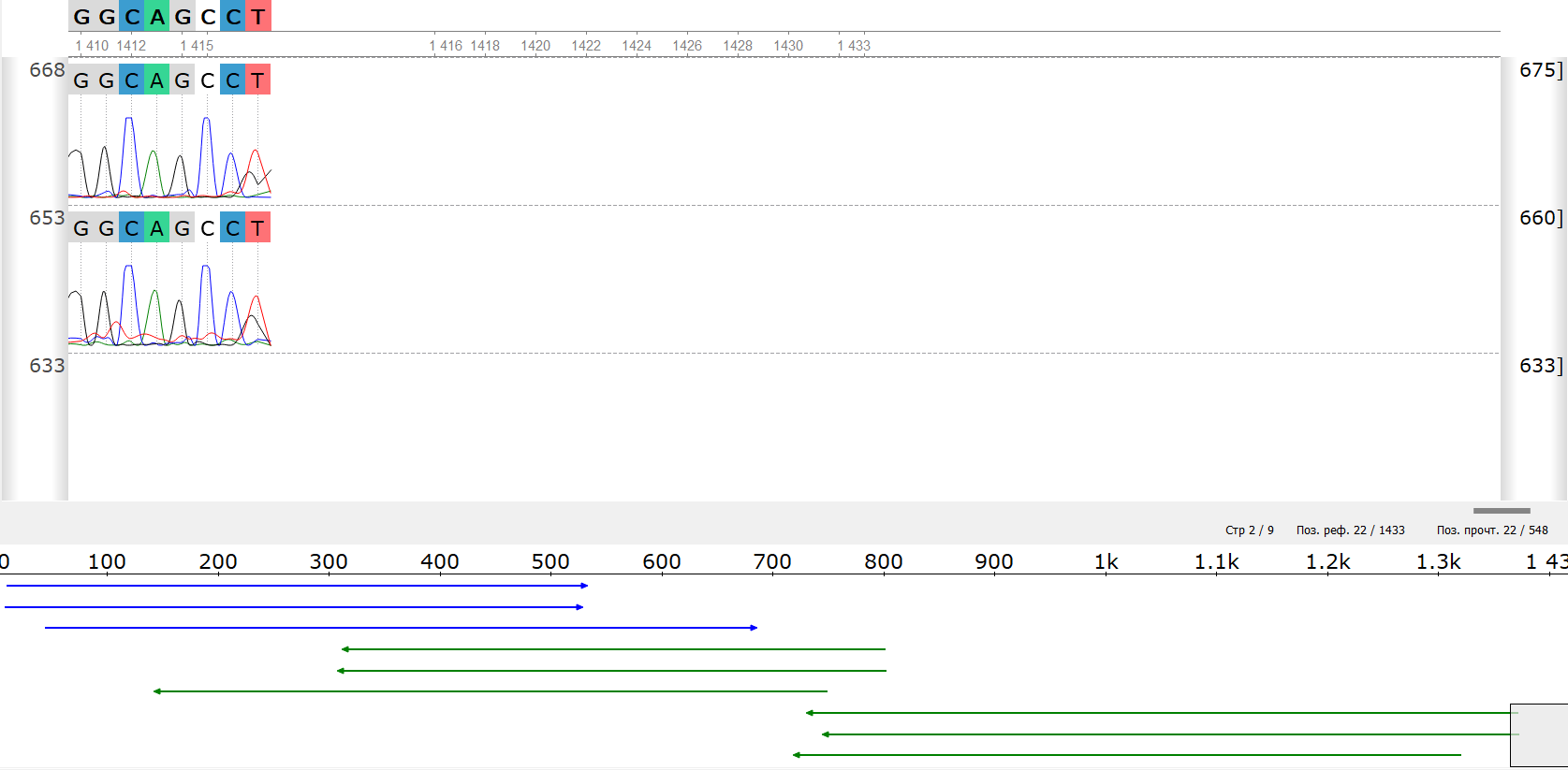
consensus

1 1492R

2 1492R

3 1492R

|  | >Consensus | Al36 |  | | | | | |
| --- | --- | --- | --- | --- | --- | --- | --- | --- |
| 1 | GGCGTGCCTA | ACACATGCAA | GTCGAGCGGT | CCACCGTTTT | GTCTGGTTTA | TACCGGGTGG | GCGGTGGATA | 70 |
| 71 | GCGGCGGACG | GGTGAGTAAC | GCGTGGATAA | CCTGCCCATT | AGACCGGGAT | AACGCTGGGA | AACTGGCGCT | 140 |
| 141 | AATACCGGAT | ACGCTCCTTG | TTCCGCATGG | GGTGAGGAGG | AAAGGGGAAA | CCCGCTAATG | GATGGGTCCG | 210 |
| 211 | CGTCCCATTA | GCTAGATGGT | GGGGTAACGG | CCTACCATGG | CGACGATGGG | TAGCCGGCCT | GAGAGGGTGA | 280 |
| 281 | CCGGCCACAC | TGGGACTGAG | ACACGGCCCA | GACTCCTACG | GGAGGCAGCA | GTGGGGAATC | TTCCGCAATG | 350 |
| 351 | GGCGAAAGCC | TGACGGAGCG | ACGCCGCGTG | AGCGAGGAAG | GCCTTCGGGT | CGTAAAGCTC | TGTTCTGGGG | 420 |
| 421 | GAAGAAGGAA | GTGACGGTAC | TCCAGGAGAA | AGCCCCGGCT | AACTACGTGC | CAGCAGCCGC | GGTAAGACGT | 490 |
| 491 | AGGGGGCGAG | CGTTGTCCGG | AATCACTGGG | CGTAAAGGGC | GCGTAGGCGG | TCCAAGAAGT | CAGTGGTGAA | 560 |
| 561 | ATACCGCAGC | TCAACTGCGG | GGGTGCCATT | GAAACCATTG | GACTTGAGGG | CAGGAGAGGG | GAGTGGAATT | 630 |
| 631 | CCCGGTGTAG | CGGTGAAATG | CGTAGATATC | GGGAGGAACA | CCAGTGGCGA | AGGCGGCTCT | CTGGCCTGGC | 700 |
| 701 | CCTGACGCTG | AGGCGCGAAA | GCGTGGGGAG | CAAACAGGAT | TAGATACCCT | GGTAGTCCAC | GCTGTAAACG | 770 |
| 771 | ATGGGTGCTA | GGTGTTGGGG | GGATAGGACC | CTTCAGTGCC | GTAGCTAACG | CAATAAGCAC | CCCGCCTGGG | 840 |
| 841 | GAGTACGGCC | GCAAGGCTGA | AACTCAAAGG | AATTGACGGG | GGCCCGCACA | AGCGGTGGAG | CATGTGGTTT | 910 |
| 911 | AATTCGACGC | AACGCGAAGA | ACCTTACCAG | GGCTTGACAT | CTCTCTGACG | TTCGTGGAAA | CACGGATTTC | 980 |
| 981 | ACGCGGGGTA | ACCCGTGTGA | CAGGGAGACA | GGTGGTGCAT | GGTTGTCGTC | AGCTCGTGTC | GTGAGATGTT | 1050 |
| 1051 | GGGTTAAGTC | CCGCAACGAG | CGCAACCCCT | ACGGTTAGTT | GCTAACGGGT | AAAGCCGAGC | ACTCTAGCCG | 1120 |
| 1121 | GACTGCCGTT | GACAAAACGG | AGGAAGGTGG | GGATGACGTC | AAATCATCAT | GCCCCTTATG | TCCTGGGCTA | 1190 |
| 1191 | CACACGTGCT | ACAATGGCCG | GTACAGAGGG | CAGCGAACCC | GTGAGGGGGA | GCGAATCCCA | AAAAGCCGGT | 1260 |
| 1261 | CTCAGTTCGG | ATTGCAGGCT | GCAACTCGCC | TGCATGAAGT | CGGAATCGCT | AGTAATCGCA | GGTCAGCATA | 1330 |
| 1331 | CTGCGGTGAA | TACGTTCCCG | GGCCTTGTAC | ACACCGCCCG | TCACACCACG | AAAGCCGACA | ACACCCGAAG | 1400 |
| 1401 | CCGGTGAGCT | AACCCTGCAA | AGGGAGGCAG | CCT |  |  |  | 1433 |

**Supplementary Figure 5.** Alignment of Sanger chromatograms of the 16S rRNA fragment sequences obtained with primers 27F, 907R, and 1492R of Al36 culture (1), cells outgrown from Al36 spores in the first experiment (2), cells outgrown from Al36 spores in the second experiment (3).
